# Efficacious Removal of Cd^2+^ and Pb^2+^ Ions from Wastewater Using a Novel Fe_3_O_4_/SiO_2_/PANI-SDBS Nanocomposite

**DOI:** 10.3390/ma18092083

**Published:** 2025-05-01

**Authors:** Mahmoud M. Youssif, Marek Wojnicki

**Affiliations:** 1 Faculty of Non-Ferrous Metals, AGH University of Krakow, al. A. Mickewicza 30, 30-059 Krakow, Poland; 2 Department of Chemistry, Faculty of Science, Tanta University, Tanta 31527, Egypt

**Keywords:** magnetic adsorbents, wastewater treatment, heavy metal removal, surface functionalization, reusable adsorbents, adsorption capacity

## Abstract

The current work synthesizes and characterizes a new Fe_3_O_4_/SiO_2_/PANI-SDBS nanocomposite designed as an efficient adsorbent for the removal of Cd^2+^ and Pb^2+^ ions from contaminated water. The process includes the polymerization of aniline on the Fe_3_O_4_/SiO_2_ nanocomposite in the presence of SDBS. The Fe_3_O_4_/SiO_2_/PANI-SDBS nanocomposite was characterized by using a variety of techniques, including FT-IR, XRD, TEM, SEM, BET, TGA, zeta potential measurements, and particle size distribution analysis, to evaluate its magnetic, structural, and surface properties. For the elimination of both Cd^2+^ and Pb^2+^ ions, ideal adsorption parameters were examined, including pH, adsorbent dose, and contact duration. The solution medium’s optimal pH for achieving the highest effectiveness of elimination for both metal ions was decided to be 7.0. The Fe_3_O_4_/SiO_2_/PANI-SDBS adsorbent demonstrated high adsorption capacities for both Pb^2+^ (72.20 mg g^−1^) and Cd^2+^ (67.84 mg g^−1^) at pH 7, with corresponding removal efficiencies of over 94.10% and 77.47%, respectively. This efficiency is attributed to the composite’s large specific surface area and the strong binding affinity of its PANI and SDBS functional groups toward heavy metal ions. Multilayer adsorption on heterogeneous surfaces was shown by isotherm analysis that matched the Freundlich model and adsorption kinetic investigations that showed strong conformance with pseudo-second order for both metal ions. The thermodynamic study proves endothermic and spontaneous process for the removal of metal ions. Furthermore, the adsorbent may be readily recovered from solution thanks to the magnetic core, and regeneration by acid treatment enables reusability with consistent adsorption efficiency across several cycles, making it a cost-effective and sustainable option for continuous water purification processes. Its high adsorption capacity and reusability also make it suitable for use in emergency-response situations, such as the rapid cleanup of wastewater.

## 1. Introduction

Heavy metal ions have become more prevalent in the environment, thus causing serious concerns in recent years, owing to their toxicity and persistence. Particularly dangerous metal ions include arsenic, cadmium, copper, chromium, mercury, nickel, and lead because of their capacity to bioaccumulate and result in serious health problems [1,2]. Mining, battery manufacturing, textiles, petroleum refining, and the paint industry are the main industrial operations that release these heavy metals into water systems [3,4]. Particularly, exposure to lead and cadmium has been connected to a number of health concerns, such as cardiovascular disease, neurological abnormalities, and renal damage. These metals also change the chemical and physical characteristics of water, which can have a negative influence on aquatic ecosystems [5,6]. According to World Health Organization (WHO) guidelines, the acceptable limits are 0.003 mg L^−^^1^ for cadmium and 0.01 mg L^−^^1^ for lead. In contrast, the Environmental Protection Agency’s maximum allowable levels are slightly higher, at 0.005 mg L^−^^1^ for cadmium and 0.015 mg L^−^^1^ for lead [7,8]. Wastewater treatment is essential for reducing water pollution because it eliminates contaminants, permits safe disposal, or makes it easier to reuse for things like industrial activities or irrigation. A number of treatment techniques are used to treat heavy metal pollution, including adsorption chemical precipitation, electrolysis, filtration, flocculation, and ion exchange [9,10,11,12]. Adsorption has been the most successful of these because of its high removal efficiency, selectivity, cost-effectiveness, operational simplicity, adaptability in targeting a variety of contaminants, and low creation of secondary pollutants [13,14]. Conventional adsorbent materials such as activated carbon, zeolites, polymers, and biomaterials have been extensively studied and evaluated for their potential in adsorbing hazardous heavy metal ions [15,16,17,18]. There is now a rising interest worldwide in developing effective adsorbents for water treatment applications, as adsorption efficiency is largely dependent on the adsorbent properties. Therefore, a large number of researchers are working to develop more effective adsorbents. Innovative nanomaterial-based adsorbents have been developed to enhance the removal of heavy metal ions from contaminated water, offering improved adsorption performance [19,20,21].

Magnetic ferrite nanocomposites, especially those based on iron oxide (Fe_3_O_4_), have become promising materials for the removal of heavy metal ions due of their enormous surface area, which enables effective adsorption of contaminants like organic compounds, dyes, and heavy metals, and their magnetic properties, which facilitate easy separation from aqueous solutions [22,23,24]. These materials can be employed directly or as a core material after being coated with appropriate functional groups for water and wastewater treatment [25]. Higher adsorption effectiveness is made possible by coating these magnetic nanoparticles with silica (SiO_2_), which increases their stability and surface area. Furthermore, the incorporation of conductive polymers like polyaniline (PANI) into the composite improves the composite’s adsorption effectiveness through chelation and electrostatic interactions with heavy metals [26,27]. Furthermore, the nanocomposite is dispersed by the surfactant sodium dodecylbenzene sulfonate (SDBS), which prevents agglomeration and increases the effective surface area, enhancing its capacity to adsorb heavy metal ions [28].

In this study, a Fe_3_O_4_/SiO_2_ nanocomposite was synthesized and subsequently integrated into a modified polyaniline using sodium dodecylbenzene sulfonate (SDBS) as a surfactant, resulting in the creation of a novel Fe_3_O_4_/SiO_2_/PANI-SDBS nanocomposite. This advanced magnetic adsorbent was designed for the effective elimination of cadmium (Cd^2+^) and lead (Pb^2+^) ions from aqueous solutions. The performance and reusability adsorption of the nanocomposite were evaluated under various influencing factors. The application of the novel Fe_3_O_4_/SiO_2_/PANI-SDBS nanocomposite as an adsorbent for water purification represents the unique approach adopted in this study.

## 2. Materials and Methods

Every chemical and reagent of analytical grade was purchased without further purification. Sigma-Aldrich supplied the tetraethyl orthosilicate (TEOS) and sodium dodecylbenzene sulfonate (SDBS). The supplier of FeSO_4_. 7H_2_O and FeCl_3_. 6H_2_O was Acros Organics. The sources of ammonium hydroxide, ammonium persulfate, and aniline were Chempur, (Piekary Śląskie, Poland). The provider of hydrochloric, sulfuric, and nitric acids was Chemland Materials (Stargard, Poland); ethanol of HPLC quality was also used. The supplier of sodium hydroxide was Avantor Performance Materials (Gliwice, Poland). PbCl_2_ and CdCl_2_. 2H_2_O, two metal salts of analytical quality, were procured from Chempur (Piekary Śląskie, Poland) and utilized for the preparation of stock solutions that included 1000 mg L^−1^ of each.

### 2.1. Synthesis of Fe_3_O_4_ Nanoparticles

**Figure 1:**
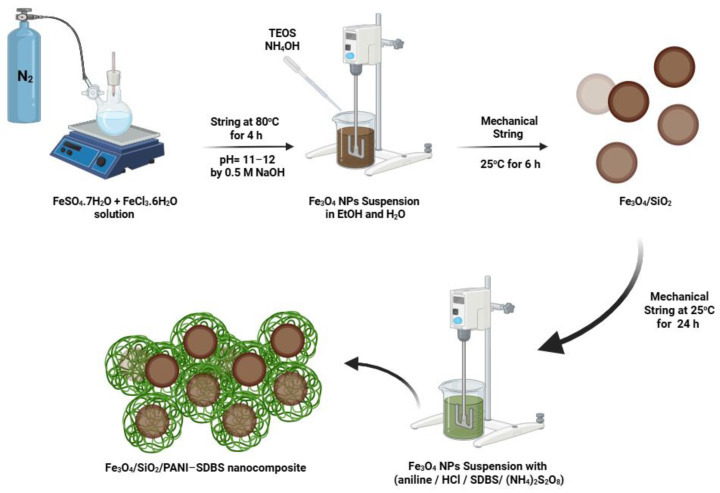
Synthesis of Fe_3_O_4_ nanoparticles, Fe_3_O_4_/SiO_2_ nanocomposite, and Fe_3_O_4_/SiO_2_/PANI-SDBS nanocomposite.

Magnetite nanoparticles were formed through the approach known as co-precipitation, using a molar ratio of 2:1 of FeCl_3_. 6H_2_O and FeSO_4_. 7H_2_O in alkaline medium [29]. Then, 0.09 mol of anhydrous FeCl_3_. 6H_2_O and 0.045 mol of FeSO_4_. 7H_2_O was dissolved in 250 mL of double-distilled water and put into a necked flask. A 0.5 M concentration of NaOH solution, which was likewise made using double-distilled water, was used to monitor the pH until it reached 11–12. N_2_ was injected into the flask to keep the environment inert during the synthesis process. After that, the reaction was conducted at room temperature with mechanical stirring (500 rpm) and heated to 80 °C for a further three hours (Figure 1). The magnetite particles were repeatedly cleaned with distilled water using magnetic extraction and then again with ethanol to produce a soft powder. Overnight, the powder dried at 80 °C.

### 2.2. Synthesis of Fe_3_O_4_/SiO_2_ Nanocomposite

The nanoparticles were dispersed in ethanol and deionized water solution containing 90 mL ethanol and 250 mL deionized water. As the solution was being stirred at 500 rpm, 5 mL of ammonia (25%) was added. Then, 3 mL of tetraethyl orthosilicate (TEOS) was added dropwise while stirring. At room temperature, the stirring continued for 6 h. The silica-coated magnetite was magnetically separated and then repeatedly cleaned with water and ethanol. Subsequently, the composite was placed in an oven set at 60 °C to dry, and then it was stored in a desiccator (Figure 1) [30].

### 2.3. Synthesis of Fe_3_O_4_/ SiO_2_/PANI-SDBS Nanocomposite

To produce the Fe_3_O_4_/SiO_2_/PANI-SDBS nanocomposite, 3 g of Fe_3_O_4_/SiO_2_ was dispersed in 7 mL of aniline and 15 mL of HCl (1 M) for 15 min. A solution of 4 g of sodium dodecylbenzene sulfonate (SDBS) in 100 mL of water was prepared. After adding SDBS to the aniline solution, the solution was sonicated for 10 min. In order to guarantee complete polymerization of aniline, 100 mL of 0.5 M ammonium persulfate solution was added to the mixture and mechanically stirred for 24 h (Figure 1). The dark green precipitate was obtained after filtering, washing several times with HCl (1 M), and drying for 12 h at 60 °C in an oven.

### 2.4. Characterization and Tools

Fourier-transform infrared spectroscopy analysis (Nicolet 380, Waltham, MA, USA) was employed to identify functional groups present in the powdered samples. The components were finely ground with 0.2 g of potassium bromide (≥99%, Sigma-Aldrich, St. Louis, MO, USA), using an agate mortar. The mixture was then pressed into pellets with a 10 mm diameter punch, using a hydraulic press capable and applying pressures up to 10 MPa. The shape, size, and distribution of the nanocomposite were analyzed using scanning electron microscope (SEM, JEOL, JCM-6000 Plus, Tokyo, Japan) and transmission electron microscope (TEM, TECNAI TF 20 X-TWIN (FEI), Hillsboro, OR, USA). For elemental analysis, SEM was coupled with energy-dispersive X-ray spectroscopy, operating at an acceleration voltage of 15.00 keV. The crystalline structure and grain size of the materials were investigated through XRD analysis using a Rigaku MiniFlex II instrument (Tokyo, Japan), equipped with Cu Kα radiation (λ = 1.54059 Å), 40 kV, and 40 mA, and scanning at 2θ = 10–70 degrees at a rate of 2 degrees/min. Thermal stability was evaluated using a thermogravimetric analyzer (DSC-TGA, TA Instruments, New Castle, DE, USA), which measured the change in material weight as a function of temperature. The temperature was raised from 25 °C to 800 °C, with an increment rate of 10 °C/min. The materials underwent thermal studies in an argon environment, with a gas flow rate of 100 mL/min, unless specified differently. Magnetic characteristics of the Fe_3_O_4_ nanoparticles and the composite were assessed with a vibrating sample magnetometer (VSM, LDJ 9600, LDJ Electronics Company, Ventura, CA, USA) operating within a magnetic field range of −8000 to 8000 Oe. The specific surface area of the nanocomposite was measured using Micromeritics ASAP 2010 equipment (Norcross, GA, USA). At 200 degrees Celsius and 4 µmHg of vacuum, the samples were degassed for 24 h. The measurement begins when the measuring cell achieves a vacuum of 10 µmHg, with five repeated measurements at 77.35 K as the relative pressure increased in 120-minute intervals. The zeta potential and particle size distribution of colloidal suspensions were evaluated using a Malvern Zeta Sizer Nano ZS (Malvern Instrument Ltd., Malvern, UK). Residual metal ion concentrations during adsorption and desorption experiments were quantified using an Atomic Emission Spectrometer (MP-AES, Agilent 4200, Santa Clara, CA, USA).

### 2.5. Adsorption Experiments

#### 2.5.1. Batch Experiment

Numerous adsorption studies have assessed the Fe_3_O_4_, Fe_3_O_4_/SiO_2_, and Fe_3_O_4_/SiO_2_/PANI-SDBS nanocomposites’ adsorption capabilities for the removal of Cd^2+^ and Pb^2+^ ions. For the experiment, stock solutions (1000 mg L^−1^) of metal ions were made using deionized water. After that, these stock solutions were diluted to give final concentrations between 5 and 60 mg L^−1^. HCl and NaOH (0.1 M) were added to solutions to assist in controlling the pH of the mixture within the range of 1 to 10. Then, 30 mg of the magnetic nanocomposite was introduced at a time to a pH = 7 solution that included 50 mL of metal ions. Following several minutes of ultrasonication, the mixture was placed on a water shaker thermostat and agitated at 120 rpm while operating at 30 °C. We recorded the time when the reaction mixture was exposed to the magnetic nanocomposite. The adsorbent was magnetically removed at certain intervals of time. MP-AES was employed to measure the concentrations of Cd^2+^ and Pb^2+^ ions in the supernatant using standard solutions of the two ions (5 to 100 mg L^−1^). Equations (1)–(3) were employed to calculate the elimination efficiency (R%) and the quantity of metal ions adsorbed at time (q_t_) and at equilibrium (q_e_).

(1) $$R\;\%=\frac{C_{o}-C_{t}}{C_{o}}⨯100\%$$

(2) $$q_{t}=\frac{{(C}_{o}-C_{t})}{m}\times V$$

(3) $$q_{e}=\frac{{(C}_{o}-C_{e})}{m}\times V$$

Initially, C_o_ is the concentration of metal ions, C_e_ is the equilibrium concentration, and C_t_ is the concentration at t. The volume of the Pb^2+^ and Cd^2+^ solution is represented by V (L), whereas the mass of the adsorbent is represented by m (g). R (%) represents the efficiency of removal, while q_t_ and q_e_ (mg. g^−^^1^) represent the adsorption capabilities of nanocomposites at equilibrium and at a specified time.

#### 2.5.2. Reusability Study

The Fe_3_O_4_/SiO_2_/PANI-SDBS nanocomposite was magnetically removed from the purified water after Cd^2+^ and Pb^2+^ adsorption. The nanocomposite was then added in 50 mL of a solution containing 0.1 M HNO_3_ and HCl with stirring for 12 h at 25 °C. The nanocomposite was repeatedly washed with distilled water after being magnetically removed from the solution after the allotted period of time. It was dehydrated for three hours at 100 °C before being reapplied for adsorption. Removing metal ions from the adsorbent was the aim of this process. Subsequent adsorption tests were conducted using the recycled Fe_3_O_4_/SiO_2_/PANI-SDBS nanocomposite under the same conditions. Five cycles of the same process were performed.

## 3. Results and Discussion

### 3.1. Characterization of Adsorbent

#### 3.1.1. FTIR Study

**Figure 2:**
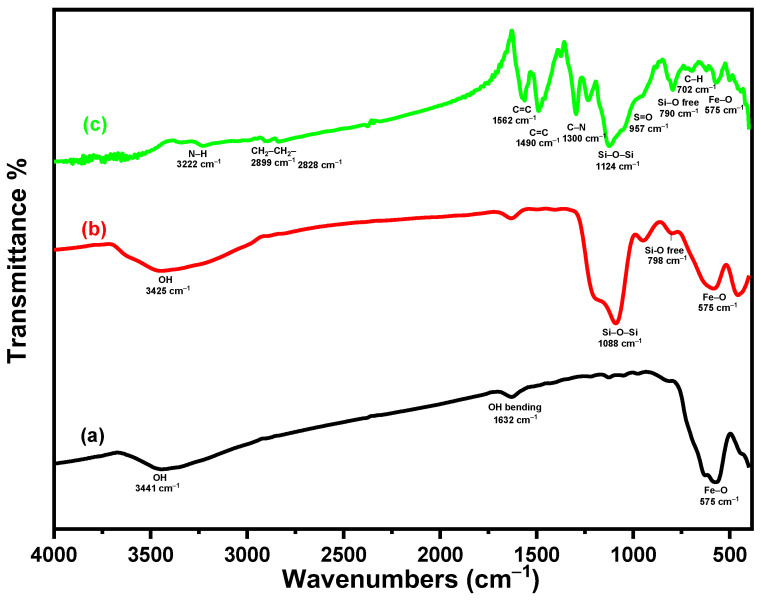
(**a**) FTIR spectra of Fe_3_O_4_, (**b**) Fe_3_O_4_/SiO_2_, and (**c**) Fe_3_O_4_/SiO_2_/PANI-SDBS nanocomposites.

Figure 2 displays the FTIR spectra of Fe_3_O_4_, Fe_3_O_4_/SiO_2_, and Fe_3_O_4_/SiO_2_/PANI-SDBS nanocomposites that were measured in the 400–4000 cm^−1^ region. A peak at 575 cm^−1^ in the Fe_3_O_4_ spectra may be attributed to the Fe–O stretching vibration band of tetrahedral and octahedral sites. Therefore, the stretching vibration band of the OH groups on the surface and the physically adsorbed water are probably accountable for the notable band at 3441 cm^−1^ [31]. The spectrum of Fe_3_O_4_/SiO_2_ exhibits three distinct peaks at 3425, 1088, and 798 cm^−1^. Each peak corresponds to the stretching vibrations of the silanol group (Si–OH) that are formed by hydrogen bonding with the surface-physiosorbed water molecules, spine siloxane bonds (Si–O–Si), and free silanol groups, respectively [32]. These results validated the existence and efficiency of the silica shell coating of the Fe_3_O_4_ nanoparticles. The peak at 3222 cm^−1^ for the Fe_3_O_4_/SiO_2_/PANI-SDBS nanocomposite is ascribed to the stretching of secondary aromatic amine (N–H) vibrations in PANI [33]. Benzenoid and quinoid units’ C=C stretching vibration is responsible for the bands at 1490 and 1562 cm^−1^ [34]. The out-of-plane and in-plane bending vibrations of C-H are responsible for the peaks at 623 and 702 cm^−1^, and the peak which at 1234 cm^−1^ corresponds to the C-H stretching vibration of the quinoid rings, whereas the C–N stretching band of an aromatic amine is seen at 1300 cm^−1^ [35,36]. Furthermore, the peaks at 2899 and 2828 cm^−1^ verified that the stretching mode was present on the long alkyl tail of SDS, and the SO_3_ group in the nanocomposite’s S=O extension is responsible for the peak at 957 cm^−1^ [37]. According to the findings, the novel Fe_3_O_4_/SiO_2_/PANI-SDBS nanocomposite was successfully formed.

#### 3.1.2. XRD Study

**Figure 3:**
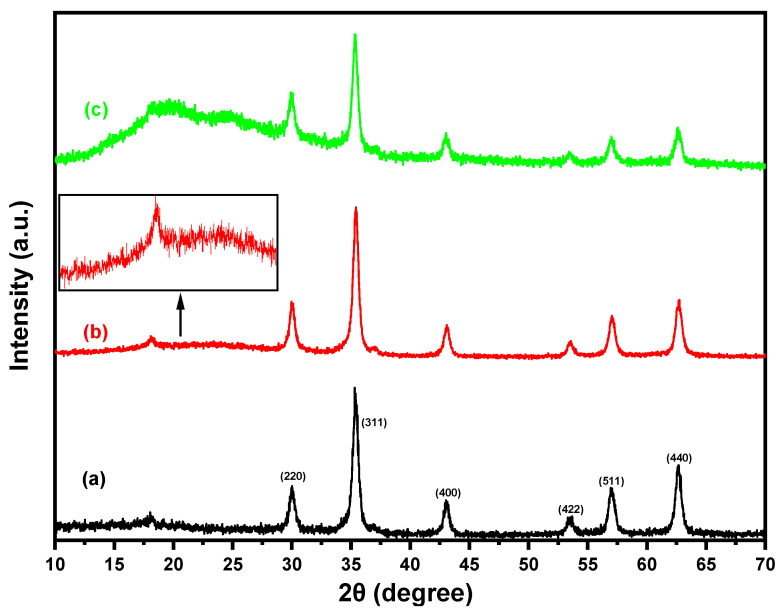
XRD patterns of (**a**) Fe_3_O_4_, (**b**) Fe_3_O_4_/SiO_2_, and (**c**) Fe_3_O_4_/SiO_2_/PANI-SDBS nanocomposites.

As seen in Figure 3, XRD analysis was employed to assess the crystallinity of the (a) Fe_3_O_4_, (b) Fe_3_O_4_/SiO_2_, and (c) Fe_3_O_4_/SiO_2_/PANI-SDBS nanocomposites. The characteristic peaks of the XRD pattern of magnetite nanoparticles at 2θ = 30.04°, 35.32°, 43.11°, 53.56°, 56.99°, and 62.67° can be ascribed to the planes (220), (311), (400), (422), (511), and (440), in that order, and can be recognized as the cubic spinel crystal structure of Fe_3_O_4_ according to (JCPDS Card No. 19-0629) [38]. Furthermore, the broad peak for Fe_3_O_4_/SiO_2_ that appeared in the region of 15° to 25° shows that Fe_3_O_4_ was successfully coated with an amorphous SiO_2_ layer [39]. Additionally, as illustrated in Figure 3c, the X-ray diffraction pattern of the Fe_3_O_4_/SiO_2_/PANI-SDBS nanocomposite shows that PANI and Fe_3_O_4_/SiO_2_ were successfully combined because the parallel vertical periodicity of the PANI chain results in the appearance of a new diffraction broad peak in the 12.94° to 30° range [40,41]. Due to its generally amorphous structure, polyaniline (PANI) may lessen the composite’s crystallinity, which would alter the intensity and sharpness of the Fe_3_O_4_ peaks. Moreover, Fe_3_O_4_/SiO_2_ and Fe_3_O_4_/SiO_2_/PANI-SDBS surfaces showed comparable characteristic peaks upon integration with PANI. This implies that the crystalline phase of Fe_3_O_4_ nanoparticles does not change in topological structure or intrinsic characteristics during the silica coating and polymerization processes [42].

XRD results were utilized to analyze the structural properties of the samples. The average crystallite size was determined using the Scherrer equation, which relates the broadening of diffraction peaks to the size of coherently diffracting domains. The equation is expressed as follows:

(4) $$\;D=\frac{K\cdot \lambda }{\beta \cdot cos\theta }$$

where D is the average crystallite size, K is the dimensionless shape factor, which is typically equal to 0.9; λ is the X-ray wavelength (Cu *Ka* radiation, λ = 0.154 nm); β is the full width at half maximum (FWHM) of the diffraction peak, corrected for instrumental broadening and expressed in radians; and θ is the Bragg angle corresponding to the diffraction peak.

The average crystallite size of the bare Fe_3_O_4_ nanoparticles was 15.04 ± 1.99 nm. After silica coating (Fe_3_O_4_/SiO_2_), it increased slightly to 15.92 ± 3.17 nm, accompanied by a notable rise in standard deviation. However, due to the overlapping uncertainty ranges and the relatively small change in mean size, this difference is not statistically significant, suggesting that the silica coating does not substantially affect the crystallite dimensions of the Fe_3_O_4_ core. Subsequent polyaniline functionalization (Fe_3_O_4_/SiO_2_/PANI-SDBS) produced a significant reduction of the crystallite size to 12.96 ±1.93 nm, which is attributed to polymer-induced lattice strain and interfacial disorder effects. The PANI coating appears to create a constraining barrier around the nanoparticles, generating structural distortions that manifest as decreased coherent diffraction domains in XRD analysis. These results demonstrate that while the silica coating preserves the core’s structural crystallinity, the conductive polymer coating introduces substantial surface modifications that alter the apparent crystallite size without necessarily changing the physical core dimensions.

#### 3.1.3. TEM and Elemental Analysis

**Figure 4:**
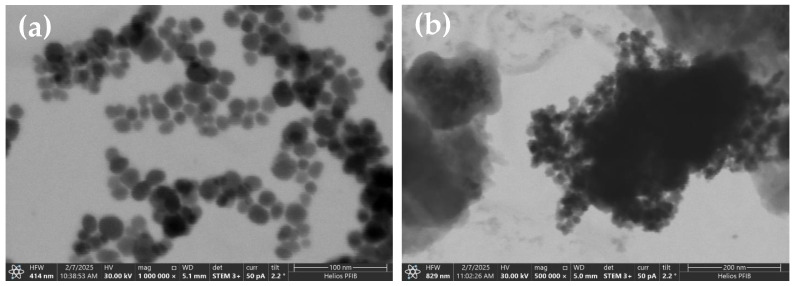
TEM images of (**a**) Fe_3_O_4_ nanoparticles and (**b**) Fe_3_O_4_/SiO_2_/PANI-SDBS nanocomposite.

**Figure 5:**
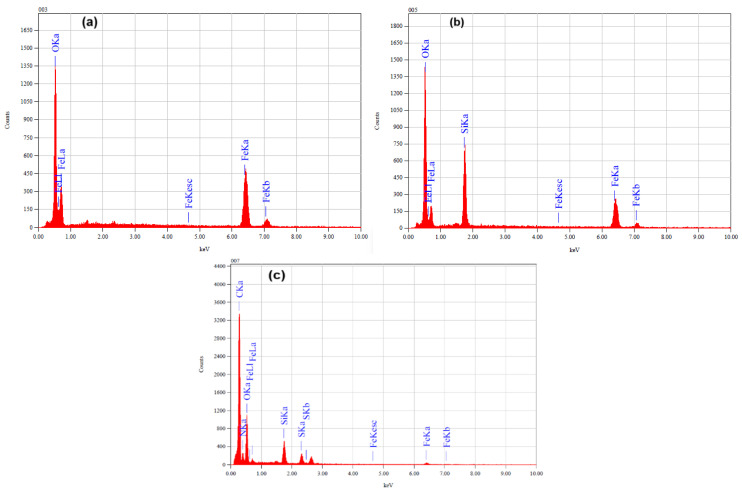
EDS analysis of (**a**) Fe_3_O_4_, (**b**) Fe_3_O_4_/SiO_2_, and (**c**) Fe_3_O_4_/SiO_2_/PANI-SDBS nanocomposites.

The TEM analysis shows an aggregation of spherical or nearly spherical Fe_3_O_4_ nanoparticles with a uniform size distribution that have particle size in the range from 10 to 25 nm, as given in Figure 4a. The particles are expected to exhibit a dense crystalline structure, which is characteristic of magnetite. Comparing the Fe_3_O_4_/SiO_2_/PANI-SDBS nanocomposite to the pure Fe_3_O_4_ nanoparticles, the TEM image shows a more complicated structure. SiO_2_ (silica), which surrounds the darker Fe_3_O_4_ core with a lighter, amorphous shell, is most likely the coating that covers the Fe_3_O_4_ nanoparticles. Nanoparticle stability and dispersibility are enhanced by this silica coating. It was anticipated that the PANI-SDBS (polyaniline functionalized with sodium dodecyl benzene sulfonate) component would surround the Fe_3_O_4_/SiO_2_ particles with a network or matrix of polymers, resulting in a heterogeneous nanocomposite structure (Figure 4b). The PANI-SDBS contributes to the material’s adsorption and conductivity qualities and may take the form of an amorphous or fibrous layer. As shown in Figure 5, Fe_3_O_4_, Fe_3_O_4_/SiO_2_, and Fe_3_O_4_/SiO_2_/PANI-SDBS nanocomposites were analyzed for their elemental compositions using energy-dispersive X-ray spectroscopy (EDS). Table 1 shows the mass percentage of each element in the prepared materials. In Figure 5a, Fe_3_O_4_ shows iron (Fe) and oxygen (O), which are consistent with magnetic nanoparticles. As shown in Figure 5b, the Fe_3_O_4_/SiO_2_ composite demonstrates a distinct silicon (Si) peak in addition to iron and oxygen, demonstrating that Fe_3_O_4_ was coated successfully. Further, in Fe_3_O_4_/SiO_2_/PANI-SDBS nanocomposites (Figure 5c), new peaks corresponding to carbon (C), sulfur (S), and nitrogen (N) are obtained from polyaniline (PANI) and sodium dodecylbenzene sulfonate (SDBS). Because nitrogen is derived from amine groups in the polymer, the peaks confirm successful functionalization of the composite with PANI. The EDX results demonstrate the step-by-step synthesis and functionalization of the nanocomposites, from Fe_3_O_4_ to Fe_3_O_4_/SiO_2_/PANI-SDBS. In environmental remediation applications, the functionalized material is suitable for adsorption and magnetic separation due to its elemental composition.

#### 3.1.4. BET Measurement

**Figure 6:**
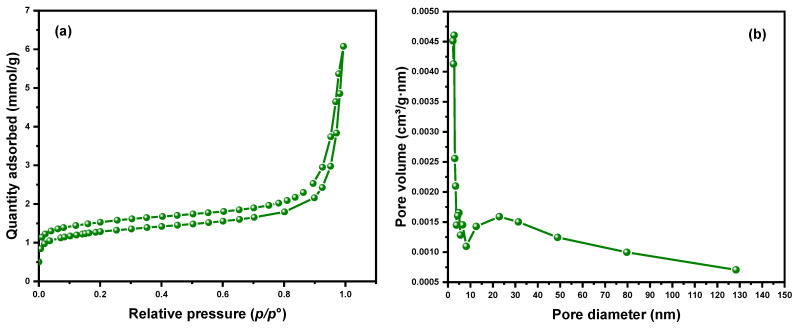
(**a**) N_2_ adsorption–desorption isotherms and (**b**) BJH pore size distribution curve of Fe_3_O_4_/SiO_2_/PANI-SDBS nanocomposites.

Several types of nanocomposites were synthesized, and their specific surface areas were measured using the BET (Brunauer–Emmett–Teller) method, which measures the adsorption of gas molecules onto a surface at specific pressures [43]. For applications such as adsorption and catalysis, this technique provides essential information about the material’s surface properties [44]. A summary of the results can be found in Table 2. According to the BET analysis, Fe_3_O_4_ nanoparticles had the lowest specific surface area of 28.71 m^2^ g^−1^. The dense and compact structure of magnetic nanoparticles accounts for this relatively small surface area. A significant increase in surface area was observed after SiO_2_ was incorporated into the Fe_3_O_4_ nanomaterial. This enhancement can be attributed to the porous nature of silica, which introduces additional surface sites and creates a more open structure. Upon further functionalization with polyaniline (PANI) doped with sodium dodecylbenzene sulfonate (SDBS), the specific surface area of the Fe_3_O_4_/SiO_2_ composite increased considerably to 116.67 m^2^ g^−1^. Silica’s inherent porosity and the polyaniline’s high surface-to-volume ratio contribute to the composite’s overall accessibility to adsorbates, thus explaining why the increase is so dramatic. Based on the BET results, each functionalization step impacts the material’s structure and increases its adsorption potential. Additionally, the porosity of the Fe_3_O_4_/SiO_2_/PANI-SDBS nanocomposite is investigated by using the N_2_ adsorption–desorption measurements that were performed at a relative pressure of 0.1–1.0, as depicted in Figure 6a. It was observed that, according to IUPAC classification, the composite displayed a type-IV isotherm with H3 adsorption hysteresis loop. The pore size distribution was also measured using the Barret, Joyner, and Halenda (BJH) method and is shown in Figure 6b. The Fe_3_O_4_/SiO_2_/PANI-SDBS nanocomposite has a pore volume of 0.17 cm^3^/g and a pore diameter of 0.67 nm, indicating the presence of a mesoporous structure. The mesoporous structures have been reported to show very high gas responses and rapid gas-responding kinetics, which are attributed to their high surface area and well-defined porous architecture, respectively [45,46]. Larger specific surface areas and porosity structures provide more sites for pollutant binding, making them more effective in adsorption processes. The high surface area and porosity structure of the Fe_3_O_4_/SiO_2_/PANI-SDBS nanocomposite make it a promising adsorbent material for removing metal ions from aqueous solutions, highlighting its effectiveness as an advanced adsorbent material.

#### 3.1.5. TGA Study

**Figure 7:**
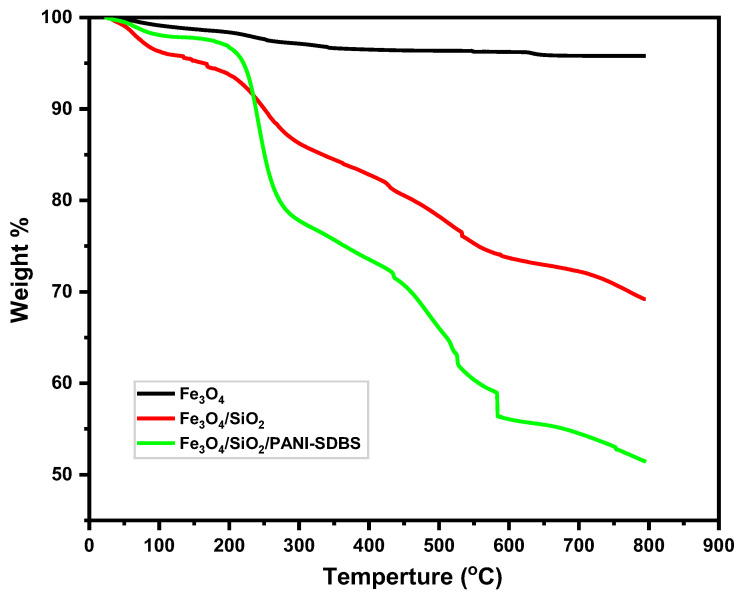
TGA analysis of Fe_3_O_4_, Fe_3_O_4_/ SiO_2_, and Fe_3_O_4_/SiO_2_/PANI-SDBS nanocomposites.

Thermal stability and decomposition behavior of Fe_3_O_4_, Fe_3_O_4_/SiO_2_, and Fe_3_O_4_/SiO_2_/PANI-SDBS nanocomposites were determined using Thermogravimetric Analysis (TGA) and are presented in Figure 7. These methods shed light on weight variations, related temperature events, and material stability. As can be seen in Figure 7, limited weight loss is shown in the TGA curve for Fe_3_O_4_, mostly as a result of the removal of moisture and surface hydroxyl groups at temperatures lower than 200 °C. It has been observed that the core Fe_3_O_4_ nanoparticle exhibits excellent thermal stability, with negligible decomposition up to 800 °C. In Fe_3_O_4_/SiO_2_, weight loss occurs in two steps: the first occurs below 250 °C, when water is lost from the adsorption surface. Second, a weight loss after 250 °C is caused by the degradation of impurities or incomplete condensation products of silica. The TGA curve of the fully functionalized nanocomposite shows more pronounced weight loss. Initially, moisture and residual solvents are lost below 200 °C. A significant weight loss occurs between 200 °C and 800 °C due to the thermal degradation of organic compounds including polyaniline and the surfactant (SDBS). Weight loss stabilizes at higher temperatures, ensuring that the composite is thermally stable for its intended applications. However, despite the functionalization of nanocomposite components that are thermally degradable, the nanocomposite remains sufficiently stable for practical applications such as wastewater treatment.

### 3.2. Adsorption Studies for Cd^2+^ and Pb^2+^ Ions

#### 3.2.1. Adsorbent Type

**Figure 8:**
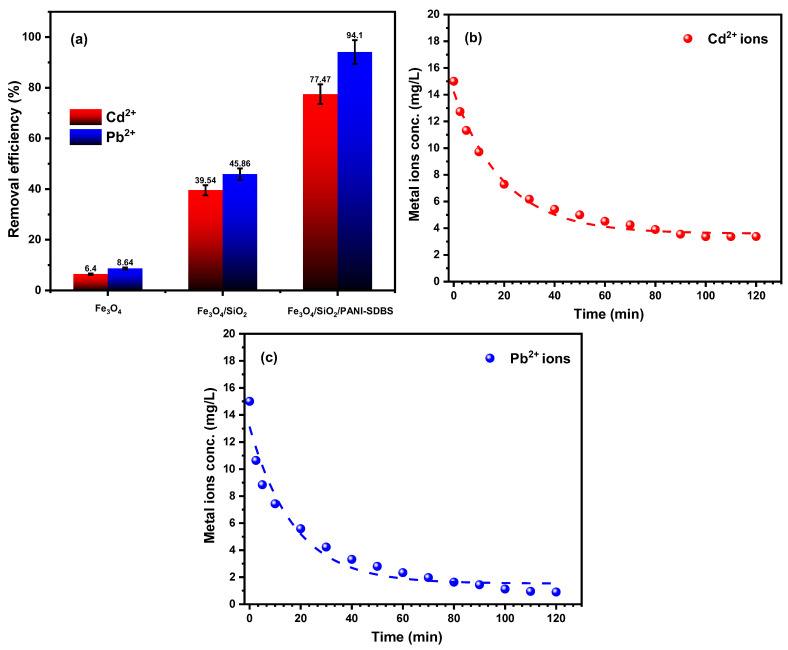
(**a**) The impact of different adsorbents on the removal of Cd^2+^ and Pb^2+^ ions. (**b**,**c**) The concentration changes in Cd^2+^ and Pb^2+^ ions in solution during adsorption on Fe_3_O_4_/SiO_2_/PANI-SDBS nanocomposite (30 mg) at pH 7.0 ± 0.1, at 30 °C.

The creation of effective adsorbents to eliminate metal ions that threaten human health and contaminate water is the primary objective of this study. The Fe_3_O_4_/SiO_2_/PANI-SDBS nanocomposite’s removal efficiency make it a new and accessible adsorbent for eliminating Cd^2+^ and Pb^2+^ ions as contaminants from wastewater. In separate trials, the nanocomposite was used to eliminate both toxic metal ions from wastewater simulations. As can be seen in Figure 8a, the study compares the efficiency of removal for Fe_3_O_4_, Fe_3_O_4_/SiO_2_, and Fe_3_O_4_/SiO_2_/PANI-SDBS towards Cd^2+^ and Pb^2+^ ions under the same conditions, having a starting metal ion concentration of 15 mg L^−1^. After 120 min, when compared to other nanomaterials, the Fe_3_O_4_/SiO_2_/PANI-SDBS nanocomposite showed the greatest removal efficacy towards Cd^2+^ ions (77.47%) and Pb^2+^ions (94.1%). Fe_3_O_4_, SiO_2_, and polyaniline were included in the nanocomposite, leading to a significant surface area increment of 116.67m²/g. This, in turn, increased the quantity of adsorption active sites, thus increasing the removal efficiency. Therefore, the Fe_3_O_4_/SiO_2_/PANI-SDBS nanocomposite was chosen to be the main adsorbent in this study for the removal of metal ions.

#### 3.2.2. Contact Time

Using 30 mg of Fe_3_O_4_/SiO_2_/PANI-SDBS adsorbent, the impact of contact duration on the Cd^2+^ and Pb^2+^ ions’ adsorption on the Fe_3_O_4_/SiO_2_/PANI-SDBS nanocomposite was examined. The percentage of metal ion elimination as a function of time is shown in Figure 8b,c. As seen in the figure, the rate of adsorption initially increased rapidly before reaching its maximum removal effectiveness for both metal ions at around 60 min. The adsorption phase achieved equilibrium when the contact duration was increased further without causing a discernible change in the equilibrium concentration. This could be as a result of the Fe_3_O_4_/SiO_2_/PANI-SDBS having a greater number of accessible empty surface sites during the first step, leading to the Cd^2+^ and Pb^2+^ ions being adsorbed on the outer surface quite fast [47].

#### 3.2.3. Nanocomposite Dose

**Figure 9:**
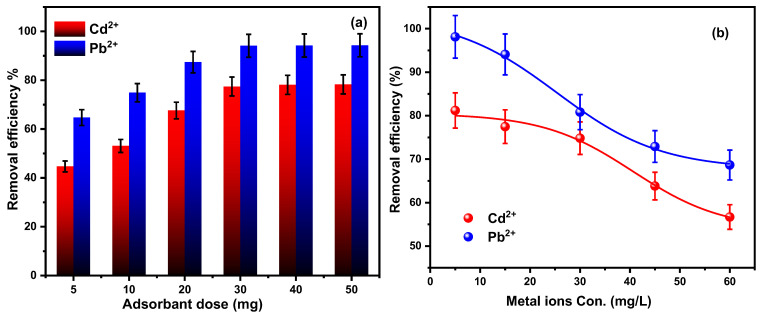
(**a**) The effect of nanocomposite dose on the removal efficiency of Cd^2+^ and Pb^2+^ ions at concentrations of 15 mg L^−1^, pH 7 ± 0.1 and 30 °C; and (**b**) Fe_3_O_4_/SiO_2_/PANI-SDBS (30 mg) adsorption efficiency for different Cd^2+^ and Pb^2+^ ion concentrations at pH 7 ± 0.1, 30 °C, and a stirring speed of 140 rpm.

The dose of adsorbent in the solution has a significant impact on the nanocomposite’s adsorption rate and efficiency. As illustrated in Figure 9a, the dose effect of the Fe_3_O_4_/SiO_2_/PANI-SDBS nanocomposite towards Cd^2+^ and Pb^2+^ ions was examined in the range of 5 to 50 mg under identical conditions of other parameters. The findings show that Fe_3_O_4_/SiO_2_/PANI-SDBS has sufficient active sites to adsorb metal ions even at low dosages and concentrations. Figure 9a shows that the adsorbed percentages of Cd^2+^ and Pb^2+^ ions increased from 44.7 to 77.4% and from 64.7 to 94.1%, respectively, when the dosage of the adsorbent rose from 5 to 30 mg. This phenomenon might be explained by the nanocomposite’s growing surface area, which leads to the availability of more adsorption positions for both metal ions [48]. The effectiveness of both metal ions’ removal stabilizes at a value of 30 mg. Furthermore, the removal efficiency for both metal ions reached a nearly constant value as the adsorbent dose rose from 40 to 50 mg, suggesting that no additional adsorption took place. This implies that equilibrium had been attained by the adsorption process [49]. Therefore, 30 mg was selected as the typical dose for this investigation.

#### 3.2.4. Metal Ion Concentration

**Figure 10:**
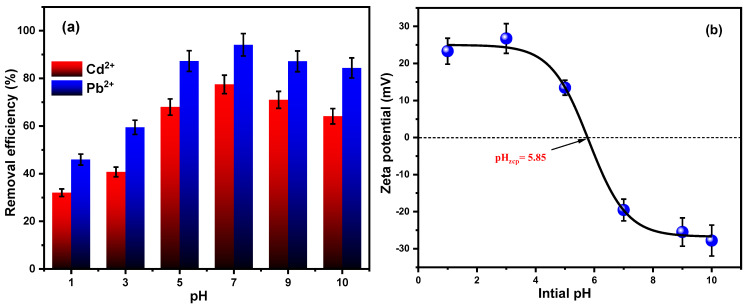
(**a**) Adsorption efficiency and (**b**) zeta potential of Fe_3_O_4_/SiO_2_/PANI–SDBS for different Ph levels.

During adsorption studies, the metal ion concentration significantly affects the adsorption rate and effectiveness. In order to assess the impacts of different concentrations of both metal ions, the tests were conducted at 30 °C with a constant nanocomposite dose (30 mg), pH = 7± 0.1, and at various metal ion concentrations ranging, from 5 to 60 mg L^−1^, as seen in Figure 9b. As seen in Figure 10b, the removal efficiency of Cd^2+^ and Pb^2+^ ions dropped from 81.1% to 56.6% and from 98.1% to 68.6%, respectively, after 120 min, as the metal ions concentration rose from 5 to 60 mg L⁻¹. Metal ion adsorption can be influenced by a wide range of factors, such as adsorbent surface properties, electrostatic interaction, surface charge, hydrogen bonding, hydrophobic and hydrophilic properties, van der Waals forces, and others [50]. Higher metal ion removal percentages result from the limited number of ions at low concentration, which is linked to the greater availability and accessibility of adsorption positions on the adsorbent surface. However, when an extra population of ions comes into contact with a finite number of adsorption positions for both metal ions, the removal effectiveness significantly decreases at high concentrations [51].

#### 3.2.5. pH and Zeta Potential

When eliminating metal ions or other contaminants from an aqueous medium, pH is an essential variable in adsorption processes. Any adsorbent’s surface charge, morphology, and ionization state can all be greatly impacted by pH; this may also affect the overall efficacy of adsorption, as well as the interactions between the ions and the adsorbent surface. Adsorption studies are therefore necessary to ascertain the adsorption capability under various pH and to optimize the pH in order to obtain the highest adsorption performance for metal ions. Figure 10a and Figure 10b both show how pH affects the adsorption removal effectiveness of Fe_3_O_4_/SiO_2_/PANI-SDBS within a pH gradient range (1–10) at the concentration of both metal ions (15 mg L^−1^), with an adsorbent dose of 30 mg.

**Figure 11:**
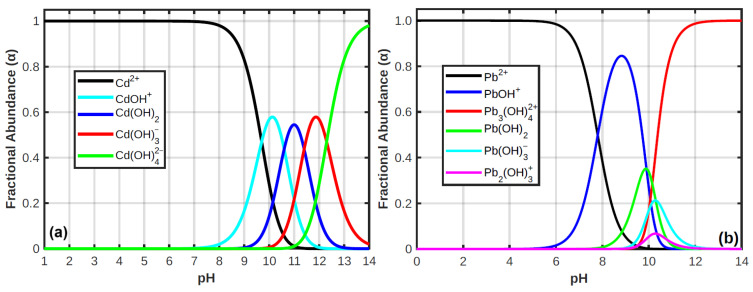
Distribution of ions in the aqueous system as a function of pH: (**a**) cadmium groups and (**b**) lead groups.

As shown in Figure 10a, the findings show that the Fe_3_O_4_/SiO_2_/PANI-SDBS nanocomposite’s removal effectiveness towards Cd^2+^ and Pb^2+^ ions reached 77.4% and 94.1%, respectively, at pH = 7. After that, the removal efficiency dropped to 64.1% and 84.4% at pH = 10. The reason for this was that at a lower pH, the Cd^2+^ and Pb^2+^ ions and hydrogen ions in the solution competed for binding with the available sites on the adsorbent’s surface, and this led to inadequate rates of removal. Additionally, it was simple to see that the zero-charge point of adsorbent is 5.85, meaning that the positively charged surface of the adsorbent created electrostatic repulsion with the Cd^2+^ and Pb^2+^ ions in the solution when pH < 5.85 [52]. In a pH-rising environment, hydrogen ions are steadily reduced, giving metal ions an advantage over hydrogen ions. Its nitrogenous functional groups also provide a soft base, which makes them attractive to softer metal ions, possibly explaining the adsorbent’s greatest removal rate at pH = 7. Nitrogenous functional groups can form stable bonds with metal ions, enhancing the adsorption process. These groups have lone pairs of electrons that can donate to the metal ions, creating physical or chemical bonds. This interaction facilitates the effective removal of metal ions from the solution [53]. After pH = 7, the adsorption efficiency decreases, most likely due to the formation of metal hydroxide species and possible precipitation, which reduces the availability of free Cd^2+^ and Pb^2+^ ions in solution for adsorption. Speciation analysis (Figure 11) confirms that at pH > 7, both metal ions begin to form hydroxy complexes or precipitates, limiting their interaction with the adsorbent surface.

Figure 10b illustrates the zeta potential of the nanocomposite across the pH range. The transition from positive to negative zeta potential as the pH increases reflects the gradual deprotonation of surface functional groups. The zero-charge point of approximately 5.85 confirms that the material carries a net-negative surface charge under neutral and alkaline conditions, thus favoring the adsorption of positively charged metal ions. PANI in the nanocomposite exhibits a well-documented pH-dependent doping/de-doping behavior due to protonation and deprotonation of its nitrogen-containing functional groups, mainly the imine groups, which are easier to be protonated than the amine groups [54]. At pH values lower than pH_PZC_, the imine groups can be protonated (doped), making the PANIs carry a positive charge, creating electrostatic repulsion with the metal ions in the solution, which limits their uptake. However, under alkaline conditions, at pH values higher than the pH_PZC_, the imine groups can be deprotonated (de-doped) as base form, resulting in reduced conductivity and negatively charged binding sites. Based on these results, it is possible to predict variation in the sorbent performances as a function of the pH of the solutions and metal ion distributions. In fact, negatively charged surfaces promote the sorption of the metal ions, whereas when the sorbents are positively charged, the removal of metal ions is promoted.

For the effect of pH of the solution, metal ion distributions were determined in different pH mediums. The results demonstrate that a majority of ionic species present in the solution are Cd^2+^ or Pb^2+^ as non-complex forms in a wide pH range (pH 1–7), from acidic to neutral medium, as shown in Figure 11. However, under alkaline conditions (pH > 7), these metal ions underwent significant transformation, primarily forming hydroxy complexes and precipitates, according to the equilibrium and the distribution of species. Overall, the optimal pH of 7 was selected for subsequent adsorption experiments because it (i) represents a neutral, environmentally relevant condition; (ii) prevents precipitation artifacts while maintaining the ionic form of Cd^2+^ and Pb^2+^; and (iii) supports favorable electrostatic conditions and functional group availability on the surface of the nanocomposite for adsorption. The combined effects of metal ion speciation, adsorbent surface charge, and PANI doping state explain the pH-dependent adsorption behavior observed.

### 3.3. Kinetics of Adsorption Studies

Kinetic isotherms provide crucial insights into the rate at which adsorption occurs, helping to understand the efficiency and mechanism of the process. They allow researchers to determine how long it will take to achieve equilibrium and the factors influencing adsorption rates. This information is essential for optimizing industrial applications, such as wastewater treatment and purification processes [55,56]. Using three linearized and nonlinearized kinetic models, the adsorption kinetics of Cd^2+^ and Pb^2+^ ions on the Fe_3_O_4_/SiO_2_/PANI-SDBS nanocomposite were investigated. Equations (5)–(9) in Table 3 reflect a pseudo-first-order, pseudo-second-order, and intraparticle diffusion model [57]. Based on the greatest regression coefficient R^2^ values, the model was evaluated according to its fitness to the data and error analysis.

**Figure 12:**
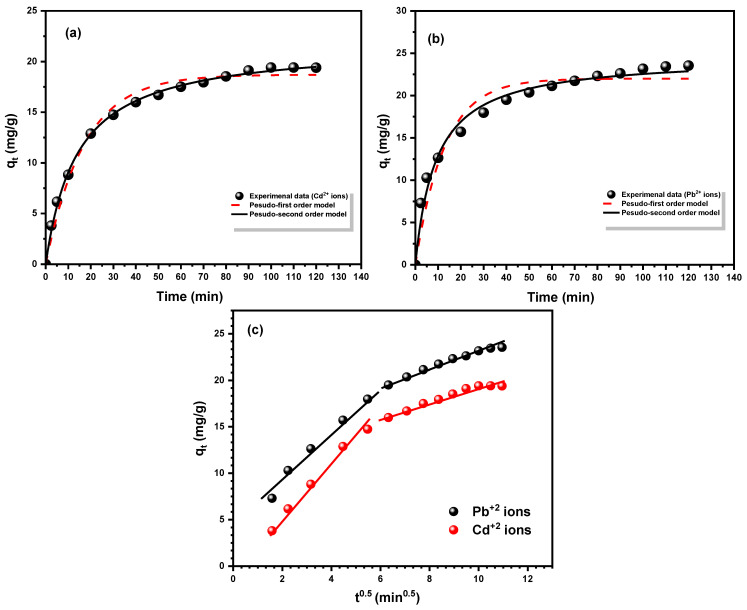
Adsorption kinetic plot of (**a**) Cd^2+^ ions and (**b**) Pb^2+^ ions on Fe_3_O_4_/SiO_2_/PANI-SDBS nanocomposites with nonlinear pseudo-first order, followed by (**c**) linear intraparticle diffusion plots for each metal ion under conditions of 15 mg L^−1^ metal ion concentration, 30 mg nanocomposite, and 50 mL metal ion solution, with 140 rpm shaking, a temperature of 30 °C, and pH = 7.0 ± 0.1.

Nonlinear curves for the pseudo-first-order and -second-order models of Cd^2+^ and Pb^2+^ ion adsorption on the Fe_3_O_4_/SiO_2_/PANI-SDBS nanocomposite are demonstrated in Figure 12a,b, respectively. Table 4 displays the calculated adsorption capacity (q_e,cal_), correlation coefficient (R^2^), and kinetic parameters for both models that were established using the data. As shown in Table 4, the pseudo-second-order model demonstrated the significant best fit to the experimental data. Therefore, the values predicted by the pseudo-second-order model and the empirically observed q_e_ values coincided. In addition, the pseudo-second-order model was selected as the most effective model to explain the adsorption of both metal ions onto the nanocomposite due to the strong connection between the experimental q_e_ values and the observed equilibrium values.

In order to further investigate the kinetics of Cd^2+^ and Pb^2+^ ion adsorption, we employed the intraparticle diffusion equation (Equation (8)). Based on the q_t_ versus t^0.5^ plot (Figure 12c), the intraparticle diffusion rate constant and the boundary layer thickness constant were determined and inserted in Table 3. This model suggests that metal ion adsorption is mostly controlled by intraparticle diffusion if the plot crosses the origin. As shown in Figure 12c, the plot here did not link to the origin, explaining that the adsorption mechanism is not the only controlling step through intraparticle diffusion; there are other steps in the adsorption process besides intraparticle diffusion that the affect adsorption rate.

Both metal ions are adsorbed on the Fe_3_O_4_/SiO_2_/PANI-SDBS composite’s surface through two stages. The initial step is mostly the consequence of boundary layer diffusion, while the second stage is related to intraparticle diffusion. The first stage has the greatest K_p_ value, whereas the second step has the lowest, according to Table 4. Because there are many adsorption sites on the surface of Fe_3_O_4_/SiO_2_/PANI-SDBS, and large concentration of metal ions, external diffusion of metal ions on the adsorbent surface happens quickly. Afterwards, metal ions started to diffuse out towards the adsorption sites inside the pores as a result of the Fe_3_O_4_/SiO_2_/PANI-SDBS surface’s adsorption sites becoming exhausted. As a result, the adsorption process was slowed down and the adsorption force decreased. In the end, the adsorption process achieves equilibrium [58].

### 3.4. Isotherm Studies

The equilibrium dispensation of adsorbate molecules between the liquid and solid phases, as well as the variation in adsorbate concentration on the surface, are quantitatively shown by adsorption isotherms. To comprehend the adsorption phenomena and precisely restrict the adsorption capacity of the Fe_3_O_4_/SiO_2_/PANI-SDBS composite, experimental data were analyzed using a variety of isotherm models. Both linear and nonlinear Freundlich, Langmuir, and Dubinin–Radushkevich (D-R) isothermal models were employed to match the Cd^2+^ and Pb^2+^ ion adsorption data on the Fe_3_O_4_/SiO_2_/PANI-SDBS nanocomposite [59,60,61].

The homogenous monolayer adsorption on the surface of nanocomposite materials was elucidated by the Langmuir isotherm. It assumes that the adsorption process occurs at energetically similar and identical active sites free from side interactions between the adsorbed molecules, even at nearby sites. Enthalpy and adsorption of activation energy are constant for all compounds [62]. The linear and nonlinear versions of this model are provided by the following equations (Equations (10) and (11), respectively [63,64,65]).

(10) $$\frac{C_{e}}{q_{e}}=\frac{1}{K_{L}q_{m}}+\frac{C_{e}}{q_{m}}$$

(11) $$q_{e}=\frac{q_{m}K_{L}C_{e}}{\left(1+K_{L}C_{e}\right)}$$

The equilibrium concentration of metal ions in the solution is shown by C_e_ (mg L^−1^). The maximum monolayer capacity is indicated by q_m_ (mg g^−1^), the Langmuir adsorption constant is K_L_, and the equilibrium adsorption capacity of Cd^2+^ and Pb^2+^ ions is represented by the variable q_e_ (mg g^−1^). The dimensionless factor, R_L_, which is displayed in Table 5, was computed using Equation (12) to assess the potential adsorption of both metal ions on the nanocomposite surface.

(12) $$R_{L}=\frac{1}{1+K_{L}C_{e}}$$

Whether the adsorption process is favorable (0 < R_L_), irreversible (R_L_ = 0), or unfavorable (R_L_ > 1) depends on the value of this component. Both Cd^2+^ and Pb^2+^ ions exhibit favorable adsorption onto the Fe_3_O_4_/SiO_2_/PANI-SDBS magnetic composite, as indicated by their respective R_L_ values of 0.298 and 0.235.

In contrast to the Langmuir model, the Freundlich model is not restricted to monolayer formation and may represent multilayer and reversible adsorption, as well as the Freundlich isotherm dealing with heterogeneous systems. Equations (13) and (14) provide the linear and nonlinear Freundlich equation, respectively, where k_F_ is the Freundlich constant (mg g^−1^), and 1/n is the adsorption intensity, which represents the degree of adsorption favorability [66].

(13) $$\ln q_{e}=\ln K_{F}+\frac{1}{n}\ln C_{e}$$

(14) $$q_{e}=K_{F}C_{e}^{1/n}$$

Both linear and nonlinear Temkin isotherm models were used to analyze the adsorption data of both metal ions on the Fe_3_O_4_/SiO_2_/PANI-SDBS nanocomposite’s surface. The adsorbent–adsorbate interaction is low in this model, and the adsorption energy of all molecules in the surface layer decreases with increased coverage of the surface [67]. Equations (15) and (16) represent this model in its linear and nonlinear version.

(15) $$q_{e}=BlnK_{T}+BlnC_{e}$$

(16) $$q_{e}=B\ln(K_{T}C_{e})$$

where the maximum binding energy is linked to the Temkin equilibrium constant, K_T_ (L mg^−1^). The formula B = RT/b is used to determine the heat of adsorption, which is represented as B (J mol^−1^). In this equation, b stands for the adsorption potential, R for the gas constant, and T for the absolute temperature in Kelvin.

Chemical and physical adsorption are distinguished through analysis of the adsorption data for both metal ions by utilizing the Dubinin–Radushkevich equations [68]:

(17) $$lnq_{e}=\ln q_{s}-\beta \varepsilon^{2}$$

(18) $$\varepsilon =RT\ln(\frac{1}{C_{e}}+1)$$

(19) $$E=\frac{1}{({2\beta )}^{0.5}}$$

Whether the adsorption mechanism is chemical or physical may be inferred from the calculated value of E from the D-R model. It follows that adsorption is considered physical when the energy (E) is less than 8 kJ mol^−1^. The term “chemical adsorption” is used when the value of E is more than or equal to 8–16 [69]. Whereas ε (kJ mol^−1^) is Polanyi potential, q_s_ (mg g^−1^) is the theoretical saturation capacity of each metal ion on the nanocomposite. The constant β is related to the adsorption energy (E, kJ mol^−1^), which is calculated using Equation (18) and listed in Table 4.

**Figure 13:**
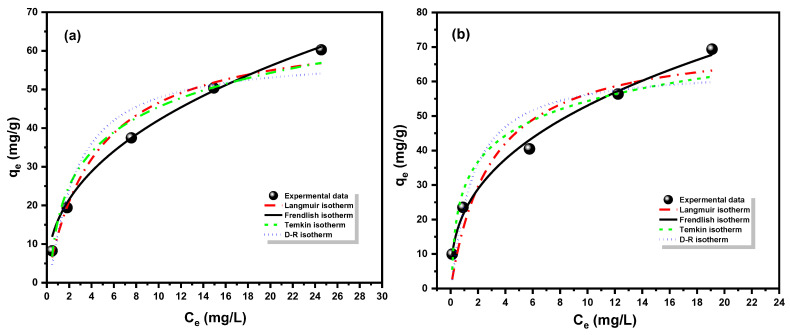
Adsorption isotherm plot of (**a**) Cd^2+^ ions and (**b**) Pb^2+^ ions on Fe_3_O_4_/SiO_2_/PANI-SDBS nanocomposite with nonlinear adsorption isotherm models for Langmuir, Freundlich, and D-R isotherm for each metal ion under conditions of 15 mg L^−1^ metal ion concentration, 30 mg nanocomposite, and 50 mL metal ion solution, with 140 rpm shaking, temperature of 30 °C, and pH = 7.0 ± 0.1.

Nonlinear adsorption of Langmuir, Freundlich, Temkin, and Dubinin–Radushkevich (D–R) isotherms is shown in Figure 13. The data obtained from the isothermal models and their correlation coefficient (R^2^) are inserted in Table 5. The adsorption data of both metal ions showed a greater correlation (R^2^ = 0.995 and 0.994) with the Freundlich model, indicating a better match. This might indicate that the surface of the nanocomposite has heterogeneous adsorption sites, which could lead to the formation of multilayers. Additionally, the values of 1/n for Cd^2+^ and Pb^2+^ ions are 0.454 and 0.364, respectively, indicating that both metal ions have favored adsorption on the Fe_3_O_4_/SiO_2_/PANI-SDBS nanocomposite’s surface. According to Table 5, Temkin isotherm experiments show that low heat of adsorption indicates weak interactions between the adsorbent and adsorbate, thus supporting the formation of hydrogen bonds and electrostatic interactions during adsorption. Furthermore, the physical adsorption of both ions on Fe_3_O_4_/SiO_2_/PANI-SDBS nanocomposite surfaces is confirmed by the low adsorption energies of Cd^2+^ and Pb^2+^ ions (Table 5).

### 3.5. Thermodynamic Study

The adsorption rate is significantly impacted by the reaction temperature. Using a constant nanocomposite dosage (30 mg) and a starting concentration of 15 mg L^−1^ of each metal ions at pH = 7 ± 0.1 and 30 °C, the effectuation of temperature on both metal ion adsorption was examined. The energy alterations linked to adsorption are fully described by the thermodynamic parameters. Three thermodynamic parameters, namely the enthalpy change (ΔH°), free energy of adsorption (ΔG°), and entropy change (ΔS°), can offer insight into the change in heat and spontaneity of the adsorption process. Equation (20)’s linear relationship between ln K_d_ and 1/T was analyzed to find the values of both ΔH° and ΔS°. Equation (21) was used to calculate the value of ΔG° in the meanwhile [70].

(20) $$\ln K_{d}=\frac{{△S}^{o}}{R}-\frac{{△H}^{o}}{RT}$$

(21) $${△G}^{o}={△H}^{o}-T{△S}^{o}$$

Here, the distribution coefficient, K_d_, was calculated according to the following equation: K_d_ = (q_e_/C_e_), where q_e_ (mg g^−1^) is the adsorbent’s adsorption capabilities at equilibrium, and C_e_ (mg L^−1^) is the equilibrium concentration of metal ions in the solution. In Equations (20) and (21), R denotes the ideal gas constant (8.314 J/mol K), and T indicates absolute temperature (K). Table 6 contains the calculated and combined thermodynamic parameters. The positive ∆H° value (25.28 and 33.57 kJ mol^−1^) for the adsorption of Cd^2+^ and Pb^2+^ ions on the Fe_3_O_4_/SiO_2_/PANI-SDBS nanocomposite, respectively, indicates that the process is endothermic. The spontaneous adsorption of Cd^2+^ and Pb^2+^ ions on the adsorbent is indicated by the negative values of ΔG° at various temperatures. However, throughout the adsorption process, the Fe_3_O_4_/SiO_2_/PANI-SDBS nanocomposite and metal ions’ interface became more random, as seen by the positive values of ΔS° (97.90 and 131.96 J mol^−1^ K^−1^) for the Cd^2+^ and Pb^2+^ ions, respectively [71].

### 3.6. Adsorbent Reusability and Regeneration Study

**Figure 14:**
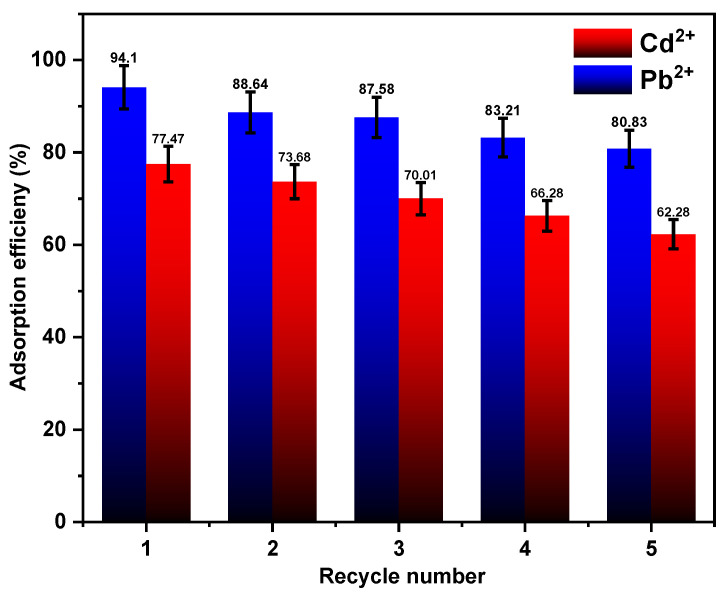
Reusability of Fe_3_O_4_/SiO_2_/PANI-SDBS nanocomposite (30 mg) Cd^2+^ and Pb^2+^ ion removal with 15 mg L^−1^ initial concentration at pH = 7.0 ± 0.1, 30 °C.

The development of economical and effective adsorbents for their ongoing usage in wastewater treatment depends on the adsorbent’s reusability, which guarantees cost-effectiveness and economic viability. At a low pH, the Fe_3_O_4_/SiO_2_/PANI-SDBS nanocomposite showed a limited adsorption capability. Consequently, the Fe_3_O_4_/SiO_2_/PANI-SDBS nanocomposite may be regenerable with acidic treatment. A strong magnet was used to remove the nanocomposite from the solution in order to evaluate its reusability. HNO_3_ and HCl (0.1 M) were used to desorb the metal ions, and the mixture was maintained at a steady temperature of 25 °C for 12 h. The adsorbent then washed three times with distilled water, separated magnetically from the solution, and dried for 24 h at 80 °C. Following that, the dried nanocomposite was used in successive adsorption cycles for each metal ion independently. The adsorption effectiveness of the nanocomposite was assessed across five iterations of adsorption and desorption (Figure 14). The removal efficiency of Cd^2+^ and Pb^2+^ decreased somewhat during the five cycles, going from 77.47 to 62.28% and from 94.10 to 80.83%, respectively, according to the results. Over five cycles, the removal effectiveness was reduced by around 15.19% for Cd^2+^ and 13.27% for Pb^2+^ ions, respectively. These results indicate the nanocomposite’s efficacy as an adsorbent for wastewater treatment. The loss of activity may be due to the hard removal of adsorbed metal ion traces from the active sites of the nanocomposite surface by the simple washing procedure. After every generation cycle, metal ions that are still present on the surface can prevent new metal ions from adhering to those active sites [72].

In addition to demonstrating effective removal and regeneration capability of the nanocomposite, using a magnetic Fe_3_O_4_ core significantly enhances the practicality of the adsorbent. The nanocomposite’s magnetic characteristic enables efficient separation from water solutions through the use of exterior magnets, which avoid filtration or centrifugation procedures. Through this feature the post-treatment process becomes easier and improves operational efficiency, especially in continuous or large-scale water purification systems. Moreover, Fe_3_O_4_ acts as a structural carrier for the SiO_2_ shell and the functional PANI-SDBS layer, contributing to the composite’s stability and uniform dispersion in solution. The combined use of magnetic separation enhances adsorbent recovery efficiency and reusability since this system proves more sustainable, as well as economical, for multiple heavy metal ion treatment cycles. The nanocomposite regeneration process offers key benefits for sustainable water treatment, including reduced material consumption, lower operational costs, and minimized environmental impact. However, the desorption method produces secondary wastewater containing metal ions, together with acid residues, while operating as a major challenge. The washing water requires appropriate treatment before being discharged. A potential solution is to recycle and reuse the desorption solution after suitable conditioning. Although this is a drawback, regeneration still offers a significant reduction in total waste compared to single-use adsorbents. Studies should focus on optimizing the washing methods to minimize the volume of regenerant used and developing environmentally friendly desorbing agents. These advancements will further enhance the eco-efficiency and industrial viability of the nanocomposite system.

### 3.7. Comparison Studies

The current adsorption maximum capacity (q_m_) of Cd^2+^ and Pb^2+^ ions by the Fe_3_O_4_/SiO_2_/PANI-SDBS nanocomposite is compared to values previously published in the literature for other adsorbents in Table 7. For Cd^2+^ and Pb^2+^ ions, the nanocomposite had an adsorption capacity of 67.84 and 73.63 mg g^−1^. Furthermore, with a percentage of 77.47% and 94.1%, respectively, it effectively removes significant amounts of Cd^2+^ and Pb^2+^ ions. These results highlight the innovative adsorbent’s encouraging potential for removing metal ions from wastewater [73,74,75,76,77,78,79,80,81,82].

### 3.8. Characterization of the Nanocomposite After Metal Ions Adsorption

#### 3.8.1. FTIR Analysis

**Figure 15:**
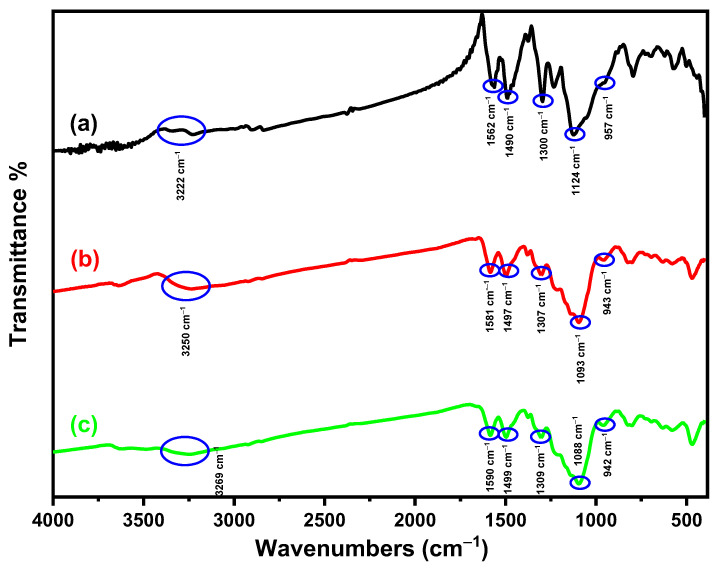
FTIR spectra of the Fe_3_O_4_/SiO_2_/PANI-SDBS nanocomposite before (**a**) and after (**b**,**c**) the adsorption of Cd^2+^ and Pb^2+^ ions, respectively.

Figure 15 illustrates the FTIR spectra of Fe_3_O_4_/SiO_2_/PANI-SDBS nanocomposite analyzed before and after adsorbed Cd^2+^ and Pb^2+^ ions to give more details on the interaction between Fe_3_O_4_/SiO_2_/PANI-SDBS and metal ions. While some spectral features remain nearly identical, several key peaks shift to higher frequencies or broaden, indicating altered electronic environments because of the polymer chain’s interaction with metal ions that decreases the extent of charge delocalization of the polymer chain. For instance, the N–H stretching vibration of secondary aromatic amine in the PANI chain shifts and becomes broad from 3222 cm⁻¹ to 3250 cm⁻¹ and 3269 cm⁻¹ after Cd^2+^ and Pb^2+^ adsorption, respectively, suggesting hydrogen bonding between the metal ions and secondary aromatic amine (N–H) of the polymer chain [83]. The peak at 1300 cm^−1^, which is related to the C–N stretching band of an aromatic amine was shifted to 1307 and 1309 cm^−1^ after adsorption of Cd^2+^ and Pb^2+^ ions adsorption, respectively, indicating the hydrogen bonding between the nanocomposite and metal ions. Similarly, the C–N stretching peak at 1300 cm⁻¹ shifted slightly to 1307 cm⁻¹ and 1309 cm⁻¹, indicating hydrogen bonding between the amine groups in the nanocomposite and the metal ions. Minor shifts in the Si–OH stretching at 1088 cm⁻¹ and S=O stretching in the SO₃ group at 957 cm⁻¹ further support the presence of weak interactions, such as hydrogen bonding and van der Waals forces, between metal ions and specific groups on the nanocomposite [84]. These spectral changes confirm that the functional groups interact with the metal ions primarily at the surface, without significant structural alteration of the nanocomposite, suggesting a physical adsorption mechanism driven by electrostatic and hydrogen-bonding interactions. This conclusion is consistent with low adsorption energy, as described by the Temkin isotherm and the monolayer adsorption indicated by the Langmuir model, reinforcing the characterization of this process as physical adsorption.

#### 3.8.2. SEM and EDS Analysis

**Figure 16:**
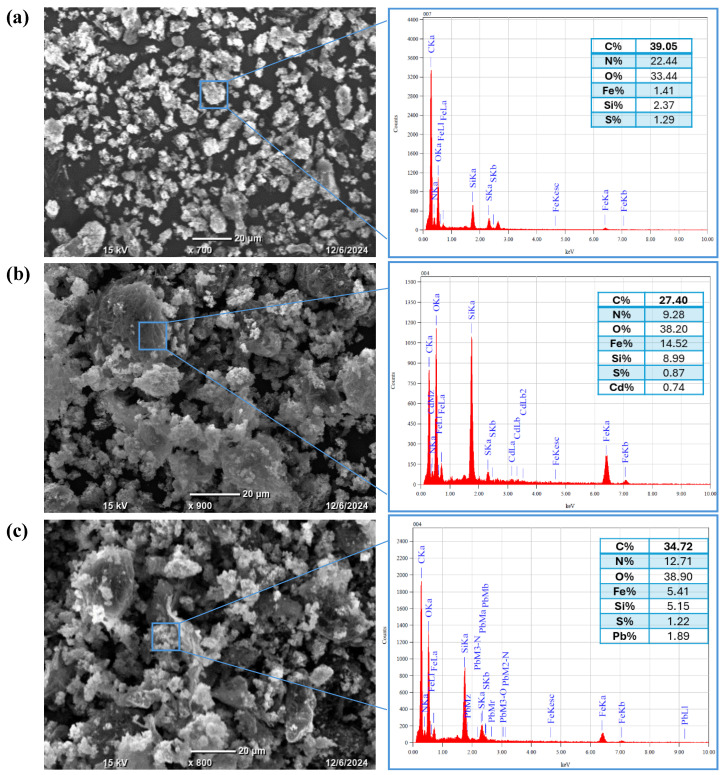
SEM/EDS of Fe_3_O_4_/SiO_2_/PANI-SDBS nanocomposite before (**a**) and after (**b**,**c**) the adsorption of Cd^2+^ and Pb^2+^ ions, respectively.

The surface morphology and elemental composition of the Fe_3_O_4_/SiO_2_/PANI-SDBS nanocomposite before and after adsorption of metal ions were analyzed using SEM-EDS analysis. Before adsorption (Figure 16a), the SEM image revealed a smooth surface with uniformly distributed particles surface. After adsorption of Cd^2+^ (Figure 16b) and Pb^2+^ (Figure 16c) ions, the nanocomposite surface showed increased roughness, surface deposits, and particle agglomeration. These morphological changes indicate the successful adsorption of the metal ions onto the active sites of the nanocomposite’s surface. EDS analysis further confirms the clear presence of Cd (0.74%) and Pb (1.89%) elements in the samples after adsorption, verifying the efficient adsorption of the metal ions on the Fe_3_O_4_/SiO_2_/PANI-SDBS nanocomposite.

### 3.9. The Proposed Adsorption Mechanism

**Figure 17:**
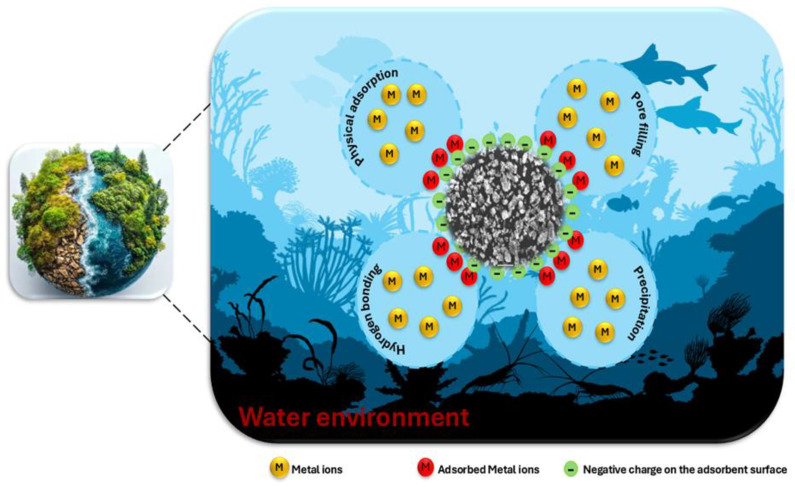
Illustration of the proposed mechanism for adsorption of metal ions on Fe_3_O_4_/SiO_2_/PANI-SDBS nanocomposite.

The adsorption mechanisms of heavy metals may be investigated to better comprehend the interactions between nanocomposite material and metal ions. This process underscores the importance of various parameters and critical factors in achieving effective removal. A variety of mechanisms, including physical adsorption, electrostatic interactions, hydrogen bonding, ion exchange, complexation, pore sorption, and redox reactions, can be used to control the adsorption of heavy metals. The adsorption process is impacted by the pH of the solution because it modifies the adsorbent’s surface charges and their interactions. As a result, the nanocomposite’s capacity to adsorb metal ions and their interactions may be enhanced or diminished. Certain groups on the surface of the Fe_3_O_4_/SiO_2_/PANI-SDBS nanocomposite, including hydroxy, amine, and sulfonated groups, can be capable of gaining or losing a proton in response to changes in the surrounding solution’s pH. To shed light on the nanocomposite’s surface properties, pH_pzc_ was determined, as shown in Figure 10b. It is about 5.85. The surface of the nanocomposite becomes positively charged overall when the pH falls below pH_pzc_ (5.85), since these groups prefer to acquire a proton. A negatively charged surface can result from these groups losing a proton when the pH rises over pH_pzc_ (5.85). Because the nanocomposite’s surface structure has negative charges, the positively charged metal ions and the negatively charged nanocomposite form hydrogen bonds and interact electrostatically, resulting in the remarkable removal effectiveness of 77.47% for Cd^2+^ and 94.10% for Pb^2+^ ions at a pH equal to 7. As shown in Figure 15, Fourier-transform infrared (FTIR) was used to examine the nanocomposite both before and after adsorption of metal ions in order to offer further details on the interaction between Fe_3_O_4_/SiO_2_/PANI-SDBS and metal ions. There are several functional groups in the Fe_3_O_4_/SiO_2_/PANI-SDBS composite’s structure, such as hydroxy, amine, and sulfonated groups, which can gain or lose a proton depending on the pH of the solution. These spectral changes confirm that the functional groups interact with the metal ions primarily at the surface without significant structural alteration of the nanocomposite, suggesting a physical adsorption mechanism driven by electrostatic and hydrogen-bonding interactions. The Fe_3_O_4_/SiO_2_/PANI-SDBS nanocomposite’s surface morphology was examined both before and after the adsorption of metal ions. Figure 16 shows how the material’s surface shape and blocks with many pores change significantly before adsorption and after the adsorption of Cd^2+^ and Pb^2+^ ions, which are indicative of physical adsorption characteristics. Physical adsorption and pore filling may be key mechanisms for heavy metal adsorption, as heavy metals may occupy these interior gaps. The surface content of Cd and Pb increased significantly after adsorption, whereas the other contents decreased, according to EDS analysis, indicating effective heavy metal ion adsorption by the nanocomposite (Figure 16). Fe_3_O_4_/SiO_2_/PANI-SDBS nitrogen adsorption–desorption isotherms and associated pore size distribution charts showed a type-IV isotherm with H3 adsorption hysteresis loop (Figure 6). The H3 hysteresis loop showed an irregular pore structure and a lack of a distinct saturation adsorption plateau, which made it easy for gas molecules to enter the pores. Using the BJH technique, the average pore diameter was found to be 0.67 nm, and the BET surface area was found to be 116.67 m^2^ g^−1^, indicating that Fe_3_O_4_/SiO_2_/PANI-SDBS has the pore characteristics that lead to a high physical adsorption capacity, and the adsorption mechanisms of all considered metal ions may involve both pore filling and pore diffusion. According to our findings, the nanocomposite surface was physically adsorbing metal ions. As shown in Figure 17, all of the data indicate that each of the functional groups is capable of eliminating metal ions through pore-filling pathways, hydrogen bonding, and electrostatic interaction.

## 4. Conclusions

The current work demonstrates the effective creation of a novel Fe_3_O_4_/SiO_2_/PANI-SDBS nanocomposite via aniline polymerization with the presence of SDBS. This nanocomposite was demonstrated to be an extremely effective adsorbent for the elimination of Cd^2+^ and Pb^2+^ ions from water. The Fe_3_O_4_/SiO_2_/PANI-SDBS nanocomposite was characterized by using a variety of techniques, including FTIR, XRD, TEM, SEM, BET, TGA, zeta potential measurements, and particle size distribution analysis to evaluate its magnetic, structural, and surface properties. To eliminate Cd^2+^ and Pb^2+^ ions, which are typical contaminants of heavy metal ions, from a simulated wastewater environment, the adsorption effectiveness of the Fe_3_O_4_/SiO_2_/PANI-SDBS nanocomposite was examined. The findings demonstrated that the nanocomposite’s superior structural and magnetic qualities make it effective at absorbing heavy metals and simple to extract from water. By optimizing factors like pH, dosage, and contact time, we observed that the optimum removal outcomes were obtained at a pH of 7.0. The structural design of the composite, which contains PANI and SDBS functional groups, allows for a high adsorption capacity for both Cd^2+^ (67.84 mg g⁻¹) and Pb^2+^ (72.20 mg g⁻¹), as demonstrated by the construction of multiple layers of adsorption on the surfaces in accordance with the Freundlich isotherm. The adsorption kinetics of these metal ions follow the pseudo-second-order model. Even under various conditions, thermodynamic investigations demonstrated spontaneous and endothermic adsorption. The magnetic core of the nanocomposite makes recovery straightforward, and with a little acid treatment, it may be successfully reused. The elimination of these metal ions was explained by a suggested mechanism. Overall, Fe_3_O_4_/SiO_2_/PANI-SDBS provides a useful, affordable way to adsorb heavy metal pollution in water, making it a good choice for wastewater treatment in the future.

## Figures and Tables

**Table 1:** EDS composition analysis of synthesized samples and mass percentage of each element.

Sample	Fe (%)	O (%)	Si (%)	N (%)	C (%)	S (%)
Fe_3_O_4_ nanoparticles	37.46	62.54	--	--	--	--
Fe_3_O_4_/SiO_2_	35.86	48.50	15.64	--	--	--
Fe_3_O_4_/SiO_2_/PANI-SDBS	39.05	33.44	2.37	22.44	39.05	1.29

**Table 2:** Specific surface area of synthesized samples.

Adsorbents	Specific Surface Area (m² g⁻¹)
Fe_3_O_4_	28.71
Fe_3_O_4_/SiO_2_	63.92
Fe_3_O_4_/SiO_2_/PANI-SDBS	116.67

**Table 3:** List of linear and nonlinear equations for pseudo-first order, pseudo-second order, and intraparticle diffusion.

Models	Form of an Equation	Equation
Linear pseudo-first order (PFO)	$\log\left(q_{e}-q_{t}\right)=\log q_{e}-\frac{k_{1}}{2.303}t$	(5)
Nonlinear pseudo-first order (PFO)	$q_{t}=q_{e}(1-e^{-k_{1}t})$	(6)
Linear pseudo-second order (PSO)	$\frac{t}{q_{t}\;}=\frac{1}{k_{2}{q_{e}}^{2}}+\frac{t}{q_{e}}$	(7)
Nonlinear pseudo-second order (PSO)	$q_{t}=\frac{k_{2}q_{e}^{2}t}{1+k_{2}q_{e}t}$	(8)
Intraparticle diffusion	$q_{t=K_{p}t^{0.5}+C}$	(9)

q_e_ and q_t_ provide the equilibrium and timely metal ion adsorption, whereas K_1_ and K_2_ are pseudo-first- and pseudo-second-order constants. C is constant, while K_P_ is the diffusion intraparticle constant for each step.

**Table 4:** Kinetic parameters for the adsorption of Cd^2+^ and Pb^2+^ ions (15 mg L^−1^) on the Fe_3_O_4_/SiO_2_/PANI-SDBS nanocomposite (30 mg) at pH 7 ± 0.1 and 30 °C.

Model	Form	Parameter	Pb^2+^ Ion	Cd^2+^ Ion
**Pseudo-first order**				
	Linear form			
		q_e.exp_ (mg/g) q_e.cal_ (mg/g) K_1_ (min^−1^) R^2^	23.53 19.55 0.091 0.937	19.39 16.82 0.091 0.956
	Nonlinear form			
		q_e.exp_ (mg/g) q_e.cal_ (mg/g) K_1_ (min^−1^) R^2^	23.53 22.99 0.078 0.938	19.39 18.70 0.058 0.983
**Pseudo-second order**				
	Linear form			
		q_e.exp_ (mg/g) q_e.cal_ (mg/g) K_2_ (g/mg min) R^2^	23.53 24.15 0.004 0.998	19.39 21.80 0.003 0.996
	Nonlinear form			
		q_e.exp_ (mg/g) q_e.cal_ (mg/g) K_2_ (g/mg min) R^2^	23.53 24.59 0.004 0.981	19.39 21.74 0.003 0.998
**Intraparticle diffusion**				
		K_p1_ (mg/g/min) C (mg/g) R^2^ K_p2_ (mg/g/min) C (mg/g) R^2^	2.64 3.87 0.98 0.88 14.13 0.978	2.85 0.136 0.988 0.78 11.27 0.944

**Table 5:** Isotherm for adsorption of Cd^2+^ and Pb^2+^ions (15 mg L^−1^) on the Fe_3_O_4_/SiO_2_/PANI-SDBS nanocomposite (30 mg) at pH 7 ± 0.1 and 30 °C.

Isotherm	Applicability Criteria	Form	Parameter	Pb^2+^	Cd^2+^
Langmuir	Single-layer or homogeneous adsorption	Linear form	K_L_ (L/mg)	0.426	0.237
			q_max_ (mg/g)	72.20	67.84
			R^2^	0.939	0.974
		Nonlinear form	K_L_ (L/mg)	0.340	0.228
			q_max_ (mg/g)	72.88	66.92
			R^2^	0.913	0.956
Freundlich	Non-uniform distribution or multilayer adsorption	Linear form	N	2.74	2.20
			K_F_ (mg/g)	22.66	14.76
			R^2^	0.994	0.995
		Nonlinear form	N	2.64	2.41
			K_F_ (mg/g)	22.18	16.15
			R^2^	0.993	0.987
Temkin	Uniform distribution or heterogeneous surface	Linear form	B_T_ (J/mol)	14.68	14.83
			K (L/mg)	10.79	2.41
			R^2^	0.901	0.966
		Nonlinear form	B_T_ (J/mol)	14.43	13.07
			K (L/mg)	10.92	3.10
			R^2^	0.904	0.974
Dubinin–Radushkevich (D-R)	Differentiate between chemical and physical absorption	Linear form	q_s_ (mg/g)	46.27	42.11
			E (J/mol)	101.8	50.61
			R^2^	0.801	0.804
		Nonlinear form	q_s_ (mg/g)	64.34	61.26
			E (J/mol)	711.96	135.88
			R^2^	0.884	0.946

**Table 6:** Thermodynamic characteristics for Cd^2+^ and Pb^2+^ ion adsorption on the Fe_3_O_4_/SiO_2_/PANI-SDBS nanocomposite at varying temperatures.

Metal Ions	ΔH° (kJ mol^−1^)	ΔS° (J mol^−1^ K^−1^)	ΔG° (kJ mol^−1^)				
			298	303	308	313	318
Cd^2+^	25.28	97.90	−3.89	−4.37	−4.86	−5.35	−5.84
Pb^2+^	33.57	131.96	−5.76	−6.42	−7.08	−7.74	−8.40

**Table 7:** Comparing adsorbents’ capacity for Cd^2+^ and Pb^2+^ ion removal from wastewater to that of different adsorbents.

Adsorbents	pH Medium	Adsorption Capacity (mg g^−1^)		Reference
		Cd^2+^	Pb^2+^	
Mesoporous alginate/β-cyclodextrin polymer beads	5	2.47	21.09	[73]
LDH-SHMP	5	24.34	45.66	[74]
PAG	7	6.29	12.49	[75]
Pectin hydrogel/Fe_3_O_4_/Bentonite	7	35.52	40.13	[76]
Sulfide-modified magnetic hydrochar described as MHC-S4	5.5	62.49	149.33	[77]
Fe_3_O_4_@SiO_2_–APTES	6	18.88	-	[78]
MnO2-modified magnetic biochar	-	18.60	49.64	[79]
Oxidized multiwalled carbon nanotubes (Ox-MWCNTs)	5	10.50	23.40	[80]
Amino/carboxylate-functionalized Fe@SiO_2_	6	62.04	--	[81]
Soybean residue–poly (acrylic acid) (SR–PAA)	6	25.76	36.75	[82]
Fe_3_O_4_/SiO_2_/PANI-SDBS	7	67.84	72.20	This work

## Data Availability

The original contributions presented in this study are included in the article. Further inquiries can be directed to the corresponding authors.

## References

[1] Rahman Z., Singh V.P. (2019). The relative impact of toxic heavy metals (THMs) (arsenic (As), cadmium (Cd), chromium (Cr)(VI), mercury (Hg), and lead (Pb)) on the total environment: An overview. Environ. Monit. Assess..

[2] Raj D., Maiti S.K. (2020). Sources, bioaccumulation, health risks and remediation of potentially toxic metal(loid)s (As, Cd, Cr, Pb and Hg): An epitomised review. Environ. Monit. Assess..

[3] Iqbal M.S., Aslam A.A., Iftikhar R., Junaid M., Imran S.M., Nazir M.S., Ali Z., Zafar M., Kanwal A., Kamil Othman N. (2023). The potential of functionalized graphene-based composites for removing heavy metals and organic pollutants. J. Water Process Eng..

[4] Jiang J., Shi Y., Ma N.L., Ye H., Verma M., Ng H.S., Ge S. (2024). Utilizing adsorption of wood and its derivatives as an emerging strategy for the treatment of heavy metal-contaminated wastewater. Environ. Pollut..

[5] Mohan D., Pittman C.U. (2007). Arsenic removal from water/wastewater using adsorbents—A critical review. J. Hazard. Mater..

[6] Ali I., Gupta V.K. (2006). Advances in water treatment by adsorption technology. Nat. Protoc..

[7] USEPA National Primary Drinking Water Regulations: EPA Limits on Cadmium and Lead. USA Environmental Protection Agency. https://www.epa.gov/ground-water-and-drinking-water/national-primary-drinking-water-regulations.

[8] WHO (2017). Guidelines for Drinking-Water Quality: Lead and Cadmium.

[9] Naushad M.u., Vasudevan S., Sharma G., Kumar A., Alothman Z.A. (2016). Adsorption kinetics, isotherms, and thermodynamic studies for Hg^2+^ adsorption from aqueous medium using alizarin red-S-loaded amberlite IRA-400 resin. Desalination Water Treat..

[10] Bashir A., Malik L.A., Ahad S., Manzoor T., Bhat M.A., Dar G.N., Hussain Pandith A. (2019). Removal of heavy metal ions from aqueous system by ion-exchange and biosorption methods. Environ. Chem. Lett..

[11] Huang Y., Wu D., Wang X., Huang W., Lawless D., Feng X. (2016). Removal of heavy metals from water using polyvinylamine by polymer-enhanced ultrafiltration and flocculation. Sep. Purif. Technol..

[12] Youssif M.M., El-Attar H.G., Hessel V., Wojnicki M. (2024). Recent Developments in the Adsorption of Heavy Metal Ions from Aqueous Solutions Using Various Nanomaterials. Materials.

[13] Bhattacharyya K.G., Gupta S. (2008). Adsorption of a few heavy metals on natural and modified kaolinite and montmorillonite: A review. Adv. Colloid Interface Sci..

[14] Rajendran S., Priya A.K., Senthil Kumar P., Hoang T.K.A., Sekar K., Chong K.Y., Khoo S.K., Ng H.S., Show P.L. (2022). A critical and recent developments on adsorption technique for removal of heavy metals from wastewater-A review. Chemosphere.

[15] Kamboj V., Tiwari D.P. (2024). Removal of heavy metal (Cu, Cr, and Ni) ions from aqueous solution using derived activated carbon from water hyacinth. Biomass Convers. Biorefinery.

[16] Buzukashvili S., Sommerville R., Hu W., Brooks O., Kökkılıç O., Ouzilleau P., Rowson N.A., Waters K.E. (2024). Zeolite synthesis from coal fly ash and its application to heavy metals remediation from water contaminated with Pb, Cu, Zn and Ni ions. Miner. Eng..

[17] Tao D., Tang Y., Zou B., Wang Y. (2024). Mesoporous Magnetic/Polymer Hybrid Nanoabsorbent for Rapid and Efficient Removal of Heavy Metal Ions from Wastewater. Langmuir.

[18] Sheraz N., Shah A., Haleem A., Iftikhar F.J. (2024). Comprehensive assessment of carbon-, biomaterial- and inorganic-based adsorbents for the removal of the most hazardous heavy metal ions from wastewater. RSC Adv..

[19] Asgharinezhad A.A., Esmaeilpour M., Afshar M.G. (2024). Synthesis of magnetic Fe3O4@SiO2 nanoparticles decorated with polyvinyl alcohol for Cu(II) and Cd(II) ions removal from aqueous solution. Chem. Pap..

[20] Thakur A., Kumar A., Singh A. (2024). Adsorptive removal of heavy metals, dyes, and pharmaceuticals: Carbon-based nanomaterials in focus. Carbon.

[21] Gomathi T., Susi S., Alam M.M., Al-Sehemi A.G., Radha E., Pazhanisamy P., Vijayakumar S. (2024). Copper(II) ion removal from aqueous solutions using alginate nanoparticles/ carboxymethyl cellulose/ polyethylene glycol ternary blend: Characterization, isotherm and kinetic studies. Polym. Test..

[22] Zohrabi Y. (2024). Synthesis and application of magnetic ferrites (MFe_2_O_4_) in the removal of heavy metals from aqueous solutions: An updated review. Mater. Sci. Eng. B.

[23] Uddin Md J., Jeong Y.-K. (2022). Adsorptive removal of pollutants from water using magnesium ferrite nanoadsorbent: A promising future material for water purification. Environ. Sci. Pollut. Res..

[24] Lourens A., Falch A., Malgas-Enus R. (2023). Magnetite immobilized metal nanoparticles in the treatment and removal of pollutants from wastewater: A review. J. Mater. Sci..

[25] Afzal S., Sherino B., Suppiah D.D., Sagadevan S., Julkapli N.M. (2024). Prospective and potential of magnetic nanoparticles in advanced and sustainable wastewater treatment. J. Water Process Eng..

[26] Zeng X., Zhang G., Zhu J., Wu Z. (2022). Adsorption of heavy metal ions in water by surface functionalized magnetic composites: A review. Environ. Sci. Water Res. Technol..

[27] Karimi Pasandideh E., Kakavandi B., Nasseri S., Mahvi A.H., Nabizadeh R., Esrafili A., Rezaei Kalantary R. (2016). Silica-coated magnetite nanoparticles core-shell spheres (Fe_3_O_4_@SiO_2_) for natural organic matter removal. J. Environ. Health Sci. Eng..

[28] Gao G., Xie S., Zheng S., Xu Y., Sun Y. (2022). Two-step modification (sodium dodecylbenzene sulfonate composites acid-base) of sepiolite (SDBS/ABsep) and its performance for remediation of Cd contaminated water and soil. J. Hazard. Mater..

[29] Lai L., Xie Q., Chi L., Gu W., Wu D. (2016). Adsorption of phosphate from water by easily separable Fe_3_O_4_@SiO_2_ core/shell magnetic nanoparticles functionalized with hydrous lanthanum oxide. J. Colloid Interface Sci..

[30] Sun L., Wu W., Yang S., Zhou J., Hong M., Xiao X., Ren F., Jiang C. (2014). Template and Silica Interlayer Tailorable Synthesis of Spindle-like Multilayer α-Fe_2_O_3_ /Ag/SnO_2_ Ternary Hybrid Architectures and Their Enhanced Photocatalytic Activity. ACS Appl. Mater. Interfaces.

[31] Abkenar S.D., Khoobi M., Tarasi R., Hosseini M., Shafiee A., Ganjali M.R. (2015). Fast Removal of Methylene Blue from Aqueous Solution Using Magnetic-Modified Fe_3_O_4_ Nanoparticles. J. Environ. Eng..

[32] Lin G., Wang H., Li X., Lai X., Zou Y., Zhou X., Liu D., Wan J., Xin H. (2018). Chestnut-like CoFe2O4@SiO2@In2O3 nanocomposite microspheres with enhanced acetone sensing property. Sens. Actuators B. Chem..

[33] Sandaruwan C., Herath H.M.P.C.K., Karunarathne T.S.E.F., Ratnayake S.P., Amaratunga G.A.J., Dissanayake D.P. (2018). Polyaniline/palladium nanohybrids for moisture and hydrogen detection. Chem. Cent. J..

[34] Noby H., El-Shazly A.H., Elkady M.F., Ohshima M. (2018). Novel preparation of self-assembled HCl-doped polyaniline nanotubes using compressed CO_2_-assisted polymerization. Polymer..

[35] Han M.G., Cho S.K., Oh S.G., Im S.S. (2002). Preparation and characterization of polyaniline nanoparticles synthesized from DBSA micellar solution. Synth. Met..

[36] Yang N., Zhai J., Wan M., Wang D., Jiang L. (2010). Layered nanostructures of polyaniline with graphene oxide as the dopant and template. Synth. Met..

[37] Chen K., He J., Li Y., Cai X., Zhang K., Liu T., Hu Y., Lin D., Kong L., Liu J. (2017). Removal of cadmium and lead ions from water by sulfonated magnetic nanoparticle adsorbents. J. Colloid Interface Sci..

[38] Hozhabr Araghi S., Entezari M.H. (2015). Amino-functionalized silica magnetite nanoparticles for the simultaneous removal of pollutants from aqueous solution. Appl. Surf. Sci..

[39] Youssif M.M., El-Attar H.G., Małecki S., Włoch G., Czapkiewicz M., Kornaus K., Wojnicki M. (2024). Mercury Ion Selective Adsorption from Aqueous Solution Using Amino-Functionalized Magnetic Fe_2_O_3_/SiO_2_ Nanocomposite. Materials.

[40] Liu P., Huang Y., Yang Y., Yan J., Zhang X. (2016). Sandwich structures of graphene@Fe3O4@PANI decorated with TiO2 nanosheets for enhanced electromagnetic wave absorption properties. J. Alloys Compd..

[41] Dong N., Zhong M., Fei P., Lei Z., Su B. (2016). Magnetic and electrochemical properties of PANI-CoFe_2_O_4_ nanocomposites synthesized via a novel one-step solvothermal method. J. Alloys Compd..

[42] Mahdavi M., Ahmad M., Haron M., Namvar F., Nadi B., Rahman M., Amin J. (2013). Synthesis, Surface Modification and Characterisation of Biocompatible Magnetic Iron Oxide Nanoparticles for Biomedical Applications. Molecules.

[43] Hasanzadeh M., Simchi A., Shahriyari Far H. (2020). Nanoporous composites of activated carbon-metal organic frameworks for organic dye adsorption: Synthesis, adsorption mechanism and kinetics studies. J. Ind. Eng. Chem..

[44] Sun M.-H., Huang S.-Z., Chen L.-H., Li Y., Yang X.-Y., Yuan Z.-Y., Su B.-L. (2016). Applications of hierarchically structured porous materials from energy storage and conversion, catalysis, photocatalysis, adsorption, separation, and sensing to biomedicine. Chem. Soc. Rev..

[45] Jia C., Zhang Y., Kong Q., Wang Q., Chen G., Guam H., Dong C. (2020). Soft-template synthesis of mesoporous NiFe2O4 for highly sensitive acetone detection. J. Mater. Sci. Mater. Electron..

[46] Li X., Peng K., Dou Y., Chen J., Zhang Y., An G. (2018). Facile Synthesis of Wormhole-Like Mesoporous Tin Oxide via Evaporation-Induced Self-Assembly and the Enhanced Gas-Sensing Properties. Nanoscale Res. Lett..

[47] Liu X., Luo J., Zhu Y., Yang Y., Yang S. (2015). Removal of methylene blue from aqueous solutions by an adsorbent based on metal-organic framework and polyoxometalate. J. Alloys Compd..

[48] Hussain S., Salman M., Youngblood J.P., Farooq U., Yasmeen S., Al-Ahmary K.M., Ahmed M. (2024). Enhanced adsorption of Congo red dye by CS/PEG/ZnO composite hydrogel: Synthesis, characterization, and performance evaluation. J. Mol. Liq..

[49] Xiang D., Zhu R., Chen Y., Zhu M., Wang S., Wu Y., Luo J., Fu L. (2024). Preparation of amidoxime modified covalent organic framework for efficient adsorption of lead ions in aqueous solution. Chem. Eng. J..

[50] Lakshmi U.R., Srivastava V.C., Mall I.D., Lataye D.H. (2009). Rice husk ash as an effective adsorbent: Evaluation of adsorptive characteristics for Indigo Carmine dye. J. Environ. Manag..

[51] Abdurahim A.A., Yahya M.D., Abdulkareem A.S., Garba U., Mustapha L.S., Zahir A., Obayomi K.S. (2024). Insightful performance analysis of fluoride ion adsorption onto graphene-zinc oxide composite beads and its prediction by Artificial Neural Network (ANN) modeling. Nano-Struct. Nano-Objects.

[52] Bo S., Luo J., An Q., Xiao Z., Wang H., Cai W., Zhai S., Li Z. (2020). Efficiently selective adsorption of Pb(II) with functionalized alginate-based adsorbent in batch/column systems: Mechanism and application simulation. J. Clean. Prod..

[53] Wu N., Guo H., Xue R., Wang M., Cao Y., Wang X., Xu M., Jang W. (2021). A free nitrogen-containing Sm-MOF for selective detection and facile removal of mercury(II). Colloids Surf. A Physicochem. Eng. Asp..

[54] Ning W., Jingjing L., Wei L., Jiangtao F., Wei Y. (2015). Synthesis of polyaniline/TiO2 composite with excellent adsorption performance on acid red G. RSC Adv..

[55] Salahshour R., Shanbedi M., Esmaeili H. (2021). Methylene Blue Dye Removal from Aqueous Media Using Activated Carbon Prepared by Lotus Leaves: Kinetic, Equilibrium and Thermodynamic Study. Acta Chim. Slov..

[56] Zuo B., Deng Q., Shao H., Cao B., Fan Y., Li W., Huang M. (2021). Fe_3_O_4_ @Mesoporous-SiO_2_ @Chitosan@Polyaniline Core–Shell Nanoparticles as Recyclable Adsorbents and Reductants for Hexavalent Chromium. ACS Appl. Nano Mater..

[57] Changwei X., Xijian L., Shimin M., Lijuan Z., Jie L. (2017). Sub-micron-sized polyethylenimine-modified polystyrene/Fe_3_O_4_/chitosan magnetic composites for the efficient and recyclable adsorption of Cu(II) ions. Appl. Surf. Sci..

[58] El-Sayed M.M.H., Elsayed R.E., Attia A., Farghal H.H., Azzam R.A., Madkour T.M. (2021). Novel nanoporous membranes of bio-based cellulose acetate, poly(lactic acid) and biodegradable polyurethane in-situ impregnated with catalytic cobalt nanoparticles for the removal of Methylene Blue and Congo Red dyes from wastewater. Carbohydr. Polym. Technol. Appl..

[59] Panda S.K., Aggarwal I., Kumar H., Prasad L., Kumar A., Sharma A., Dai-Viet N.V., Van Thuan D., Mishra V. (2021). Magnetite nanoparticles as sorbents for dye removal: A review. Environ. Chem. Lett..

[60] Bo L., Gao F., Shuangbao, Bian Y., Liu Z., Dai Y. (2021). A novel adsorbent Auricularia Auricular for the removal of methylene blue from aqueous solution: Isotherm and kinetics studies. Environ. Technol. Innov..

[61] Temkin M.J., Pyzhev V. (1940). Recent Modifications to Langmuir Isotherms. Acta. Phys. Chim..

[62] Saeed M., Munir M., Nafees M., Shah S.S.A., Ullah H., Waseem A. (2020). Synthesis, characterization and applications of silylation based grafted bentonites for the removal of Sudan dyes: Isothermal, kinetic and thermodynamic studies. Microporous Mesoporous Mater..

[63] Popoola L.T. (2019). Nano-magnetic walnut shell-rice husk for Cd(II) sorption: Design and optimization using artificial intelligence and design expert. Heliyon.

[64] Weber W.J., Morris J.C. (1963). Kinetics of Adsorption on Carbon from Solution. J. Sanit. Eng. Div..

[65] Langmuir I. (1918). The adsorption of gases on plane surfaces of glass, mica and platinum. J. Am. Chem. Soc..

[66] Freundlich H. (1907). Über die Adsorption in Lösungen. Zeitschrift für Physikalische Chemie. J. Geosci. Environ. Prot..

[67] Belhaj A.F., Elraies K.A., Alnarabiji M.S., Abdul Kareem F.A., Shuhli J.A., Mahmood S.M., Belhaj H. (2021). Experimental investigation, binary modelling and artificial neural network prediction of surfactant adsorption for enhanced oil recovery application. Chem. Eng. J..

[68] Dubinin M.M. (1989). Fundamentals of the theory of adsorption in micropores of carbon adsorbents: Characteristics of their adsorption properties and microporous structures. Carbon.

[69] Nandiyanto A.B.D., Santiuly Girsang G.C., Maryanti R., Ragadhita R., Anggraeni S., Fauzi F.M., Sakinah P., Puji Astuti A., Usdiyana D., Fiandini M. (2020). Isotherm adsorption characteristics of carbon microparticles prepared from pineapple peel waste. Commun. Sci. Technol..

[70] Özcan A., Öncü E.M., Özcan A.S. (2006). Kinetics, isotherm and thermodynamic studies of adsorption of Acid Blue 193 from aqueous solutions onto natural sepiolite. Colloids Surf. A: Physicochem. Eng. Asp..

[71] Kassimi A.E., Achour Y., El Himri M., Laamari M.R., El Haddad M. (2021). High Efficiency of Natural Safiot Clay to Remove Industrial Dyes from Aqueous Media: Kinetic, Isotherm Adsorption and Thermodynamic Studies. Biointerface Res. Appl. Chem..

[72] Lu K., Wang T., Zhai L., Wu W., Dong S., Gao S., Mao L. (2019). Adsorption behavior and mechanism of Fe-Mn binary oxide nanoparticles: Adsorption of methylene blue. J. Colloid Interface Sci..

[73] Hassan M., Naidu R., Du J., Qi F., Ahsan M.A., Liu Y. (2022). Magnetic responsive mesoporous alginate/β-cyclodextrin polymer beads enhance selectivity and adsorption of heavy metal ions. Int. J. Biol. Macromol..

[74] Hossain M.T., Khandaker S., Bashar M.M., Islam A., Ahmed M., Akter R., Alsukaibi A.K.D., Hasan M.M., Alshammari H.M., Kuba T. (2022). Simultaneous toxic Cd(II) and Pb(II) encapsulation from contaminated water using Mg/Al-LDH composite materials. J. Mol. Liq..

[75] Liu Y., Meng Y., Qiu X., Zhou F., Wang H., Zhou S., Yan C. (2023). Novel porous phosphoric acid-based geopolymer foams for adsorption of Pb(II), Cd(II) and Ni(II) mixtures: Behavior and mechanism. Ceram. Int..

[76] Maleki S.T., Beigi P., Babamoradi M. (2023). Synthesis of pectin hydrogel /Fe3O4/Bentonite and its use for the adsorption of Pb (II), Cu (II), and Cd (II) heavy metals from aqueous solutions. Mater. Sci. Eng. B.

[77] Zhang Y., Qu J., Yuan Y., Song H., Liu Y., Wang S., Tao Y., Zhao Y., Li Z. (2022). Simultaneous scavenging of Cd(II) and Pb(II) from water by sulfide-modified magnetic pinecone-derived hydrochar. J. Clean. Prod..

[78] Takanlou L.K., Farzadkia M., Mahvi A.H., Esrafili A. (2020). Heavy metal adsorption efficiency magnetic porous composites Fe_3_O_4_–SiO_2_–APTES. Desalination Water Treat..

[79] Maneechakr P., Mongkollertlop S. (2020). Investigation on adsorption behaviors of heavy metal ions (Cd^2+^, Cr^3+^, Hg^2+^ and Pb^2+^) through low-cost/active manganese dioxide-modified magnetic biochar derived from palm kernel cake residue. J. Environ. Chem. Eng..

[80] Šolić M., Maletić S., Isakovski M.K., Nikić J., Watson M., Kónya Z., Rončević Z. (2021). Removing low levels of Cd(II) and Pb(II) by adsorption on two types of oxidized multiwalled carbon nanotubes. J. Environ. Chem. Eng..

[81] Gao J., Liu X., Ren P., Gao J., Chen Y., Chen Z. (2022). Removal behavior and mechanism of amino/carboxylate-functionalized Fe@SiO_2_ for Cr(VI) and Cd(II) from aqueous solutions. Environ. Sci. Pollut. Res..

[82] Zhang M., Yin Q., Ji X., Wang F., Gao X., Zhao M. (2020). High and fast adsorption of Cd(II) and Pb(II) ions from aqueous solutions by a waste biomass based hydrogel. Sci. Rep..

[83] Dastgerdi Z.H., Meshkat S.S., Esrafili M.D. (2019). Enhanced adsorptive removal of Indigo carmine dye performance by functionalized carbon nanotubes based adsorbents from aqueous solution: Equilibrium, kinetic, and DFT study. J. Nanostructure Chem..

[84] Humelnicu D., Valentin Soroaga L., Arsene C., Humelnicu I., Iulian Olariu R. (2019). Adsorptive Performance of Soy Bran and Mustard Husk Towards Arsenic (V) Ions from Synthetic Aqueous Solutions. Acta Chim. Slov..

